# The mosquito *Aedes aegypti *has a large genome size and high transposable element load but contains a low proportion of transposon-specific piRNAs

**DOI:** 10.1186/1471-2164-12-606

**Published:** 2011-12-15

**Authors:** Peter Arensburger, Robert H Hice, Jennifer A Wright, Nancy L Craig, Peter W Atkinson

**Affiliations:** 1 Center for Disease Vector Research, Institute for Integrative Genome Biology, and Department of Entomology, University of California, Riverside, CA 92521, USA; 2 Department of Molecular Biology & Genetics and Howard Hughes Medical Institute, Johns Hopkins School of Medicine, Baltimore, MD 20742,USA

## Abstract

**Background:**

The piRNA pathway has been shown in model organisms to be involved in silencing of transposons thereby providing genome stability. In *D. melanogaster *the majority of piRNAs map to these sequences. The medically important mosquito species *Aedes aegypti *has a large genome size, a high transposon load which includes Miniature Inverted repeat Transposable Elements (MITES) and an expansion of the piRNA biogenesis genes. Studies of transgenic lines of *Ae. aegypti *have indicated that introduced transposons are poorly remobilized and we sought to explore the basis of this. We wished to analyze the piRNA profile of *Ae. aegypti *and thereby determine if it is responsible for transposon silencing in this mosquito.

**Results:**

Estimated piRNA sequence diversity was comparable between *Ae. aegypti *and *D. melanogaster*, but surprisingly only 19% of mosquito piRNAs mapped to transposons compared to 51% for *D. melanogaster*. *Ae. aegypti *piRNA clusters made up a larger percentage of the total genome than those of *D. melanogaster *but did not contain significantly higher percentages of transposon derived sequences than other regions of the genome. *Ae. aegypti *contains a number of protein coding genes that may be sources of piRNA biogenesis with two, *traffic jam *and *maelstrom*, implicated in this process in model organisms. Several genes of viral origin were also targeted by piRNAs. Examination of six mosquito libraries that had previously been transformed with transposon derived sequence revealed that new piRNA sequences had been generated to the transformed sequences, suggesting that they may have stimulated a transposon inactivation mechanism.

**Conclusions:**

*Ae. aegypti *has a large piRNA complement that maps to transposons but primarily gene sequences, including many viral-derived sequences. This, together the more uniform distribution of piRNA clusters throughout its genome, suggest that some aspects of the piRNA system differ between *Ae. aegypti *and *D. melanogaster*.

## Background

Research into the genome-wide regulation of transposons in model organisms such as *Drosophila melanogaster*, zebrafish and mice has revealed the importance of two small RNA pathways for controlling their movement thereby preserving genome stability [1-8]. This is especially important considering the abundance and diversity of transposons in eukaryote genomes in which unregulated movement of active elements, or the non-autonomous sequences they can re-mobilize, would lead to insertion mutagenesis throughout the genome resulting in a decrease in genetic fitness. In *D. melanogaster *the Piwi-interacting RNAs (piRNAs) appear to function primarily in the regulation of transposons in both the germ line and in somatic tissues that envelope the ovaries although it is clear that transposons are not the only genomic targets of piRNAs [9-13]. In addition, endo-siRNAs have been shown to target transposons in somatic tissues [10]. However, the *D. melanogaster *genome has a relatively small transposon load (only 3.86% of the 120 Mb euchromatic DNA and 77% of 24 Mb of sequenced heterochromatic DNA making an average of 15.8% across 144 Mb of the sequenced genome)[14,15] compared with many other organisms. It is not clear how similar the transposon regulatory mechanisms between it and insects with much larger genome sizes and higher transposon loads might be.

The mosquito *Aedes aegypti *has a genome size of 1.38 Gb of which nearly half (47%) is composed of transposons [16]. It is a vector of several human pathogens, most notably RNA viruses responsible for dengue and yellow fever, and so is an insect pest of high medical importance. *Ae. aegypti *is somewhat amenable to modern genetic analysis through the use of transposon-mediated genetic transformation, site-specific recombinases and RNAi leading to the emergence of novel control strategies based on the manipulation of its genome [17-20]. A curiosity is that, while genetic transformation of *Ae. aegypti *has been achieved using the exogenous transposons *piggyBac*, *Mos1 *and *Hermes*, none of these appear to re-mobilized at a frequency which allows the implementation of transposon-based genetic strategies such as gene tagging, and gene and enhancer trapping [21-24]. Indeed the failure of *piggyBac *to retain even somatic activity in transgenic lines of *Ae. aegypti *in which the *piggyBac *transposase is expressed is in contrast to the use of *piggyBac *as an incisive genetic tool in *D. melanogaster*, *Triobolium castaneum *and mice in which its mobility properties allow the identification of genes and regulatory sequences based on function [24-27]. Understanding the basis of the inactivity of these exogenous transposons in *Ae. aegypti *is important since the ability to use these (or other transposons) as mutagens is preventing the implementation of transposon-based genetic screens used to identify genes and sequences based on function. One possible explanation for the immobility of these three exogenous transposons once they have integrated into the *Ae. aegypti *genome is that they are rapidly silenced by the host's small RNA system.

Our knowledge of small RNA silencing of transposons in insects is based on studies from *D. melanogaster*. Two pathways, the piRNA and endo-siRNA pathway, are involved however many aspects of how these pathways actually function remain unknown. The endo-siRNA pathway is dicer-dependent and generates 21 nt small RNAs that target transposons in somatic and germ line tissues, including the follicle cells of the ovary [10,12]. They are also generated in Kc and S2 cells as well as the heads of adult flies and so are most likely dispersed through the somatic tissue of the insect [10].

The piRNA pathway generates small RNAs that are typically 24-30 nt in length and in *Drosophila *appears to be mainly devoted to the silencing of transposable elements ([1,2,6-8,28,29]. In *D. melanogaster *it requires the action of three genes, *Piwi*, *aubergine *(*aub*) and *Argonaute 3 *(*Ago3*), the expression of the later two being confined to the germ line [6,30]. The piRNA pathway itself is proposed to consist of two pathways, the primary piRNA pathway and the amplification or ping-pong pathway, the later acting in germ line tissues [31,32]. In *D. melanogaster *the primary piRNA pathway utilizes antisense transcripts generated from chromosomal clusters of piRNA target sequences or transposons that are then loaded onto the Piwi protein or, to a lesser extent the Aub protein, the later acting in the germ line. These piRNAs are 2'-*O*-methylated and act as guides to transcripts generated from either invading transposons or transposons (or genes) located in the clusters, thereby achieving suppression of transposition [33,34]. The ping-pong pathway requires the action of the AGO3 and Aub proteins with sense transcripts derived from piRNA target sequences or transposons being loaded onto the AGO3 protein where their 3'ends are also 2'-*O*-methylated. Antisense transcripts originating from the same loci are loaded onto the Aub protein. Working in concert, both complexes are then capable of recognizing and slicing transcripts arising from target sequences and, at the same time, generating piRNAs that fuel subsequent amplification cycles. The outcome is an effective means by which transposons are silenced in the germ line thereby increasing genome stability. The presence of piRNA clusters in the genome also provides a memory of transposon invasions of the genome that is preserved in the female germ line and so can provide some level of immunity to the subsequent invasion of transposons recognized by the incumbent piRNA machinery [9,35]. However in *D. melanogaster *it is believed that this immunity takes more than one generation to develop before it affords resistance to at least some transposons [35]. Cytotype regulation of *P *transposon transposition has been proposed to be controlled by, in part, the generation of piRNAs to the *P *transposon [35,36].

Interestingly the *Ae. aegypti *genome contains an expansion of the Piwi gene family with there being a single *Ago3 *gene and six *Piwi *genes [37]. Our analysis of *Ae. aegypti Piwi1 *indicates that it is a truncated gene and so may not be functional. It is not possible to exactly discern which *Piwi *corresponds to *Drosophila Aub *although, based on sequence similarity, we believe *Piwi2 *is the most likely candidate. *Ae. aegypti *contains single *dicer 2*, *ago 2 *and *dicer 1 *genes but two *ago1 *genes. By inference from studies in *D. melanogaster *these most likely play roles in the siRNA (*dicer 2 *and *ago2*) and miRNA (*dicer 1 *and *ago1*) pathways [38].

We wished to determine the piRNA complement of *Ae. aegypti *and to examine whether this small RNA regulatory pathway may be responsible for the control of transposons in this mosquito, whether piRNAs were generated from a few large clusters as they are in *D. melanogaster*, and whether piRNAs are also generated to protein coding genes. We report the results of sequencing seven *Ae. aegypti *small RNA libraries from five *Ae. aegypti *lines (including four transgenic lines) and one *D. melanogaster *library using high-throughput sequencing. We show that piRNA sequences are generated from piRNA clusters and from certain protein coding genes. Remarkably for an organism with such a high transposon load we show that a much lower percentage of *Ae. aegypti *piRNAs map to transposons than in *D. melanogaster*. Indeed the majority of piRNAs appear to be targeted to protein coding genes, some of which are of viral origin.

## Results

### Library descriptions

We sequenced seven *Ae. aegypti *libraries from five different mosquito lines (two lines were sequenced twice) as well as a single *D. melanogaster *library (Table 1). Four of these lines were transgenic and contained the *Hermes*, *Mos1 *or *piggyBac *transposase placed under the control of either the *Ae. aegypti *ß2-tubulin promoter or the *D. melanogaster *hsp70 promoter [23,39-41]. These four transgenic strains were designed and constructed for the separate purpose of determining whether these transposases could, using a jumpstarter strategy, remobilize their target transposons. All three experiments failed to detect significant levels of remobilization [21,23,41] (Smith and Atkinson, unpublished). The *Hermes *and *Mos1 *expressing strains were constructed in 2007 and we estimated that they had each been maintained for approximately 35 generations under selection before RNA was obtained from them for small RNA library construction. Both the *Hermes *and *Mos1 *tranposase strains were generated in the Orlando strain of *Ae. aegypti *maintained at UC Riverside. The *piggyBac *line was generated in the Liverpool strain of *Ae. aegypti*, which is also the reference genomic strain [16]. The *D. melanogaster *strain was transgenic for the same autonomous *Hermes *transposon present in the auto-*Hermes *257 *Ae. aegypti *transgenic line and had been maintained at UC Riverside since 2002. The starting material for all libraries was whole tissue adults since we wished to determine the small RNA complement directed to transposons from both germ line and somatic tissues.

**Table 1:** *Ae. aegypti *and *D. melanogaster *lines used in this study

Library number	Species	Strain	Line (transformation plasmid)	Tissue type
1	*Ae. aegypti*	Orlando	pMos3DB2Her	whole adults

4	*Ae. aegypti*	Orlando	pMos3DB2Her	whole adults

2	*Ae. aegypti*	Orlando	pBac3EB2Mos	whole adults

6	*Ae. aegypti*	Orlando	pBac3EB2Mos	whole adults

10	*Ae. aegypti*	Orlando	wild type	whole adults

11	*Ae. aegypti*	Orlando	auto-Hermes 257	whole adults

12	*Ae. aegypti*	Liverpool	pMos3DBhspPBac	whole adults

14	*D. melanogaster*	csW+	auto Hermes	whole adults

**Figure 1:**
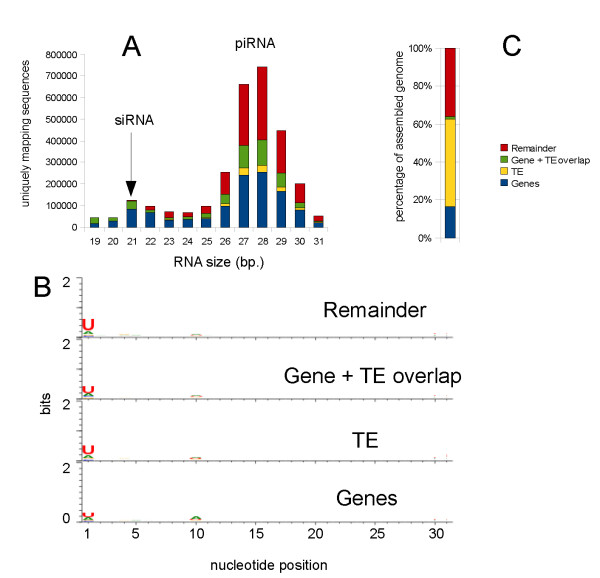
**The size distribution and assignment of piRNAs relative to genome complexity in *Ae. aegypti***. **A) **Size distribution of *Ae. aegypti *small RNA abundance in a representative library (library 1). The number of small RNAs mapping to *Ae. aegypti *genes, transposons, both, or neither, are shown as different colors for each size class (the legend is shown on the right). **B) **Relative distribution of nucleotide abundance at *Ae. aegypti *small RNA positions for small RNAs targeted to transposons, genes and other sequences. The graph was drawn using using Weblogo [78]. **C) **Percentage of the sequenced *Ae. aegypti *genome occupied by genes, transposon, both, or neither (the legend is shown on the right).

While there were some differences in total sequencing size between *Ae. aegypti *libraries, all produced similar size distribution patterns after removal of sequences matching ribosomal RNAs and miRNAs (Figures 1A, Additional file 1 Figure S1). Both *Ae. aegypti *and *D. melanogaster *libraries had sharp peaks at 21 nt and broader peaks between 24 and 31 nt. The 21 nt peaks matched previously described siRNA peaks in *D. melanogaster *[9,42]. The second peak was slightly shifted between the *Ae. aegypti *and *D. melanogaster *libraries. In *D. melanogaster *this peak was centered around 25 nt while in *Ae. aegypti *it was centered around 28 nt, the same distribution as seen for piRNAs in *Bombyx mori *and *Danio rerio *[5,43] (Additional file 1, Figure S1). The 25 nt centered peak in *D. melanogaster *matched the previously reported piRNA peak of this species [6]. The *Ae. aegypti *small RNAs between 24 - 31 nt exhibited bias for U at their 5' end and was observed for these small RNAs targeting transposons, gene and remaining sequences further supporting that these were likely to be piRNAs (Figure 1B).

Previously, 42% of *D. melanogaster *piRNAs from ovary libraries were reported to match transposon sequences and our whole adult tissue *D. melanogaster *library yielded a similar percentage (50%)[6]. However only 19% of *Ae. aegypti *piRNAs matched known transposons despite these occupying some 47% of its genome (Table 2 and Figure 1C). In both *D. melanogaster *and *Ae. aegypti *the vast majority of transposon-matching piRNAs mapped inside gene boundaries (Figure 1A) with transposon-matching piRNAs in *Ae. aegypti *mapping predominantly to the anti-sense transcription strand (72% of all piRNAs and 69% of uniquely mapping piRNAs). A similar pattern has been reported for *D. melanogaster *[7,44].

**Table 2:** Overlap of individual *D. melanogaster *piRNA clusters from Brennecke i. (2007) with one or more clusters from the present study

Top piRNA clusters Brennecke et al. (2007)					piRNA clusters from present study				
**Cluster id ^1^**	**Chr**.	**start**	**stop**	**Cluster Unique piRNAs ^2^**	**Cluster id ^1^**	**Chr**.	**start**	**stop**	**Cluster Unique piRNAs ^2^**

1	2R	2144349	2386719	1686	1	2R	2110440	2217150	2350
					2	2R	2304581	2394124	1975

2	X	21392175	21431907	986	24	X	21390160	21406447	231
					37	X	21424709	21429775	194

3	4	1258473	1348320	684	27	4	1250143	1289828	217

5	2L	20148259	20227581	482	22	2L	20136345	20153079	235
					14	2L	20164387	20176739	333
					12	2L	20198129	20230518	392

6	3L	23273964	23314199	228	9	3L	23251131	23313773	520

7	U	4015849	4029971	176	7	U	4022887	4029272	834

8	X	21505666	21684449	170	4	X	21496075	21510974	1046
					8	X	21526569	21543427	520
					33	X	21631078	21672693	206
					69	X	21604048	21622362	123

9	X	21759393	21844063	155	53	X	21786358	21878191	150

10	U	5766708	5772171	133	1246	U	5760625	5774759	6

11	3R	27895169	27905030	107	N/A ^3^	3R	N/A	N/A	N/A

12	3LHet	1402377	1557939	102	148	3LHet	1438260	1446518	55
					99	3LHet	1485097	1535498	84
					163	3LHet	1557765	1579687	50

13	3LHet	2011004	2180268	86	75	3LHet	2068714	2118742	110
					133	3LHet	2126549	2158540	62
					3480	3LHet	2167493	2167493	1

14	U	7542733	7545114	84	236	U	7532042	7545038	33

15	3LHet	238123	332969	71	N/A	3LHet	N/A	N/A	N/A

^1^Clusters were ordered and numbered according to the abundance of cluster unique piRNAs in both studies. Cluster 6 from Brennecke et al. (2007) was not reported here because it was not located on an assembled portion of the published *D. melanogaster *BGDP 5 genome.

^2^"Cluster unique piRNAs" were defined as piRNA sequences that mapped in only one location in the genome and inside a cluster.

^3^"N/A" indicates the absence of any overlapping piRNA cluster in present study.

**Figure 2:**
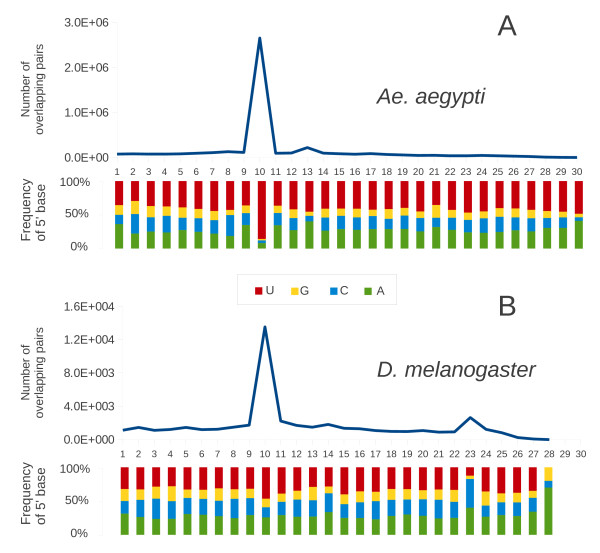
**Size distribution and frequency of 5' base of overlapping small RNAs (small RNAs at least 24 nt long, overlapping pairs on opposite strands) for all combined *Ae. aegypti *libraries (A) and the *D. melanogaster *library (B)**. The length of overlap is shown on the horizontal axes. Indicated above each axis is the number of possible overlapping pairs of small RNAs (individual small RNA sequences may be involved in multiple pairs) with specified overlap size. Indicated below each axis is the relative frequency of the 5' base identity for sequences involved in overlapping pairs. The color code for bases is indicated in the center box.

To determine if some of these small RNAs contained the 10 bp overlap ping-pong signature seen for piRNAs specific to the *D. melanogaster *germ line we plotted the distance between the 5' ends of complementary small RNAs present in *Ae. aegypti*. We also performed the same analysis on small RNAs from our *D. melanogaster *library and found that 13.5% of these piRNAs contained this ping-pong signature, a number less than the 20% reported previously from libraries prepared from dissected ovaries, the difference most likely being due to ovaries being enriched for germ line specific piRNAs with this overlap [6]. In *Ae. aegypti *the same 10 bp overlap was also found in 19.5% of piRNAs that map to opposite strands and have at least one common nucleotide position, however the proportion of piRNAs with U at the first position was higher in *Ae. aegypti *than seen in piRNAs obtained from our *D. melanogaster *library (Figure 2). These data suggest that a biochemical mechanism at least similar in function to the ping-pong amplification loop characterized in *D. melanogaster *also exists in *Ae. aegypti *however our data do not enable us to comment on its tissue-specificity nor on the proteins specifically involved in its generation.

### piRNAs in Ae. aegypti are modified

**Figure 3:**
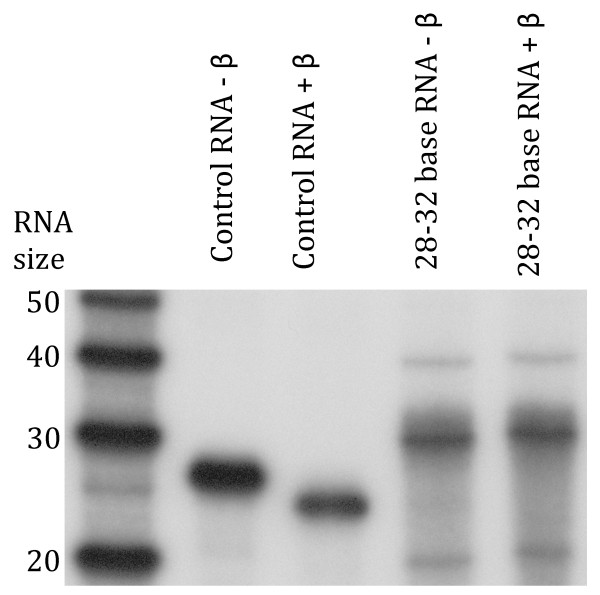
**Presumed piRNAs from *Ae. aegypti *are modified at their 3' ends. Control synthetic 23-mer RNA and purified 28-32 nt RNAs from *Ae. aegypti *were subjected to periodate oxidation and β-elimination reactions and run on a denaturing polyacrylamide gel**. Synthetic 23-mer control RNA gained mobility, as expected for an RNA not modified at its 3' end while presumed piRNAs from *Ae. aegypti *failed to gain mobility, consistent with these RNAs being modified at their 3' ends.

In both *D. melanogaster *and mice piRNAs the 3' terminal ribonucleotide contains a 2'-*O*-methyl modification that occurs after loading onto the Piwi proteins, a process catalyzed by the dmHEN1/Pimet protein [33,34]. Small RNAs containing this modification are resistant to periodate oxidation and ß-elimination [7,33]. We purified small RNAs 28-31 nt in size and performed ß-elimination on them following periodate treatment and saw no change in their mobility, suggesting that their 3' ends were modified, consistent with them being piRNAs (Figure 3).

### Estimating piRNA abundance in Ae. aegypti

**Figure 4:**
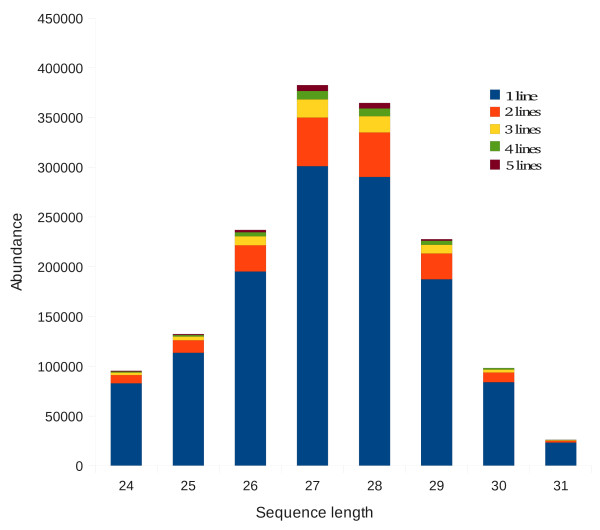
**The abundance of piRNAs derived from five *Ae. aegypti *lines (libraries from replicate lines were collapsed) by size class**. Each size class is divided into the number of piRNA sequences found in libraries derived from 1, 2, 3, 4, or 5 lines (the legend is shown at top right).

To our knowledge a single estimate of total piRNA abundance in an organism has been published to date: it being estimated that the mouse genome contains a pool 2 x10^5 ^potential piRNA sequences [45]. We attempted to perform similar estimates for the *Ae. aegypti *libraries. In order to minimize the chances that sequencing artifacts might strongly affect our results we limited our estimates to piRNA sequences that matched the published *Ae. aegypti *genome along their entire length without mismatches. Using these criteria our *Ae. aegypti *libraries contained 1,563,634 unique piRNA sequences. Furthermore, the majority of these sequences were only found in one of the five mosquito lines (Figure 4) suggesting that our sequencing efforts had sampled only a portion of a much larger piRNA pool. Betel *et al*. (2007) estimated the size of the mouse piRNA pool by extrapolating from the size of the sequenced libraries and the amount of overlap between library pairs. Applying a similar methodology (see Methods), we estimated the size of the *Ae. aegypti *piRNA pool to 1.7 × 10^7 ^(minimum estimate 5.5 × 10^6^, maximum estimate 2.3 × 10^7^) potential piRNA sequences. We also estimated the size of the piRNA pool in *D. melanogaster *based on eight published small RNA libraries derived from *Drosophila *ovaries [46]. This yielded a similar estimate of a pool of 1.6 × 10^7 ^(minimum estimate 3.9 × 10^6^, maximum estimate 2.2 × 10^7^) piRNA sequences in *D. melanogaster*.

We conclude that the pool of different *Ae. aegypti *piRNA sequences was two orders of magnitude larger than in mice but found no evidence that it was different in size from *D. melanogaster*. We note the mouse and *Ae. aegypti *libraries were not derived from the same tissue types (the mouse libraries were derived from testes), but barring a very large difference between piRNAs in mouse testes and other mouse tissues, this should not fundamentally affect our conclusions.

### piRNA Clusters

#### Validating cluster discovery methodology using the *D. melanogaster *library

piRNA clusters are believed to be the biogenesis sites of many piRNAs. *D*. *melanogaster *and *D. virilis *are to date, the only insects in which the location of piRNA clusters within their genomes has been published [9,31,47] (Brennecke 2007, Malone et al. 2009, Rozkov et al. 2010). piRNA clusters are typically identified by mapping the location of sequenced piRNA from ovary libraries to the genome assembly sequence. We sought to determine if we could find the location of such clusters using our whole tissue libraries.

Restricting ourselves to uniquely mapping piRNAs (to unambiguously identify the origin of each piRNA in the genome), we first scanned all 4,758 *Ae. aegypti *supercontigs individually using a 5 kb sliding window and identified those windows that had ten or more piRNA sequences mapping to them. Identified windows were merged if they were found adjacent to each other. The boundaries of putative cluster loci were identified by scanning for the location of the furthest piRNA sequence on either end of the locus.

While this approach was similar to that used by Brennecke *et al*. (2007), it differed methodologically in two important ways. First, we did not collapse non-contiguous windows that were less than 20 kb apart. We reasoned that this should produce smaller but more well defined loci (i.e. with few regions of low piRNA density inside the cluster). Second, because of the much larger piRNA dataset available for *Ae. aegypti*, we assigned a cut-off of more than ten uniquely mapping piRNAs per window before making that window part of a cluster. Had this cut-off not been assigned, the resulting piRNAs clusters would cover a much larger portion of the genome, but would contain very few additional piRNAs. We validated this approach by applying it to our transgenic *D. melanogaster *library and compared the resulting piRNA cluster locations to published *D. melanogaster *clusters (Table 3)[6].

**Table 3:** The top 30 piRNA clusters in *Ae. aegypti*

Cluster ID	Supercontig	start	stop	cluster size (kbps.)	cluster unique piRNAs	Distance to nearest cluster (kbps.) ^1^	Relative proportion of piRNA sequences mapping to clusters ^3^							% cluster reads unique to one library ^3^
1	supercont1.478	490638	568444	78	116,897	N/A	*23%*	*10%*	*20%*	*10%*	*9%*	*20%*	*9%*	77%

2	supercont1.1	1151499	1278727	127	94,452	75	*22%*	*12%*	*19%*	*11%*	*7%*	*20%*	*8%*	80%

3	supercont1.286	1260644	1444752	184	89,740	113	*25%*	*9%*	*23%*	*8%*	*8%*	*19%*	*7%*	82%

4	supercont1.943	196392	264127	68	21,576	N/A	*27%*	*8%*	*24%*	*8%*	*8%*	*17%*	*7%*	82%

5	supercont1.209	235416	285783	50	19,243	36	*26%*	*10%*	*23%*	*9%*	*8%*	*17%*	*7%*	82%

6	supercont1.20	3110107	3224809	115	18,814	81	*23%*	*13%*	*20%*	*12%*	*6%*	*21%*	*5%*	83%

7	supercont1.402	860224	953549	93	17,519	181	*22%*	*13%*	*20%*	*12%*	*6%*	*21%*	*5%*	83%

8	supercont1.1145	61909	94822	33	13,440	38	*25%*	*11%*	*22%*	*9%*	*8%*	*20%*	*5%*	80%

9	supercont1.435	926765	972082	45	10,856	588	*20%*	*15%*	*18%*	*13%*	*6%*	*23%*	*6%*	83%

10	supercont1.379	15854	108077	92	9,550	285	*23%*	*13%*	*21%*	*13%*	*6%*	*21%*	*4%*	85%

11	supercont1.555	368190	374829	7	8,823	80	*22%*	*15%*	*18%*	*13%*	*6%*	*20%*	*6%*	77%

12	supercont1.209	305189	366980	62	7,140	40	*24%*	*10%*	*22%*	*10%*	*7%*	*22%*	*5%*	83%

13	supercont1.109	91700	129810	38	7,083	36	*24%*	*11%*	*23%*	*11%*	*7%*	*20%*	*6%*	85%

14	supercont1.697	504405	552871	48	6,382	N/A	*30%*	*6%*	*28%*	*7%*	*9%*	*15%*	*6%*	85%

15	supercont1.164	1210657	1248495	38	6,154	35	*28%*	*12%*	*24%*	*12%*	*10%*	*6%*	*9%*	84%

16	supercont1.83	1415166	1433185	18	5,799	74	*19%*	*14%*	*17%*	*13%*	*8%*	*22%*	*7%*	83%

17	supercont1.41	2610658	2644427	34	5,422	48	*21%*	*16%*	*18%*	*16%*	*7%*	*14%*	*8%*	84%

18	supercont1.2	275115	329629	55	4,663	42	*18%*	*18%*	*16%*	*18%*	*6%*	*17%*	*6%*	86%

19	supercont1.38	3097699	3148574	51	4,653	51	*16%*	*18%*	*15%*	*18%*	*5%*	*22%*	*6%*	85%

20	supercont1.192	1700025	1763125	63	4,631	44	*18%*	*18%*	*14%*	*19%*	*6%*	*20%*	*7%*	86%

21	supercont1.38	2995043	3039699	45	4,629	31	*20%*	*14%*	*17%*	*13%*	*7%*	*20%*	*8%*	84%

22	supercont1.109	1912714	1927564	15	4,339	395	*19%*	*9%*	*12%*	*8%*	*18%*	*17%*	*17%*	92%

23	supercont1.194	895141	974760	80	4,320	53	*17%*	*19%*	*14%*	*20%*	*5%*	*20%*	*5%*	86%

24	supercont1.226	725419	748642	23	4,280	93	*21%*	*14%*	*17%*	*13%*	*7%*	*21%*	*7%*	84%

25	supercont1.518	151663	158105	6	4,164	18	*19%*	*15%*	*17%*	*15%*	*7%*	*20%*	*7%*	82%

26	supercont1.38	2572763	2684616	112	4,119	69	*17%*	*19%*	*14%*	*20%*	*5%*	*19%*	*6%*	87%

27	supercont1.90	907728	931721	24	4,004	29	*14%*	*20%*	*12%*	*19%*	*5%*	*25%*	*5%*	85%

28	supercont1.2	1423334	1464722	41	3,928	41	*20%*	*15%*	*17%*	*14%*	*7%*	*21%*	*7%*	86%

29	supercont1.192	551266	584318	33	3,859	25	*19%*	*15%*	*16%*	*15%*	*6%*	*22%*	*7%*	84%

30	supercont1.1	1420282	1453931	34	3,827	39	*24%*	*13%*	*21%*	*13%*	*6%*	*16%*	*6%*	84%

^1 ^Distances are from the center of one cluster to the next. Only cluster with 100 cluster unique piRNAs or more we considered. N/A indicates that no other piRNA cluster was found on this supercontig.

^2 ^In italics are the relative number of uniquely mapping piRNAs from each *Ae. aegypti *library. From left to right each number represents, library 1, 2,4,6,10, 11, and 12.

^3 ^Percentage of all the reads mapping to the cluster that were derived from only one of the 7 *Ae. aegypti *libraries

As expected many of the clusters defined by Brennecke *et al*. (2007) were found as two or more smaller clusters in our analysis. Nevertheless, there was overall a substantial amount of overlap between our analysis and these previously reported piRNA clusters. All but two of the top clusters were recovered (we did not recover clusters id11 and 15 reported by Brennecke *et al*. (2007)). A possible explanation may be that the piRNAs that map to these clusters are specific to germ line tissues and so were relatively underrepresented in our library. Nevertheless, because we were able to recover most of the same piRNA clusters as Brennecke *et al*. (2007) we considered our methodology sufficiently validated for analysis of our *Ae. aegypti *libraries.

### piRNA clusters in Ae. aegypti

We used the seven *Ae. aegypti *libraries to independently identify piRNA clusters from each library. All seven analyses broadly agreed on the location of the top piRNA clusters on the *Ae. aegypti *supercontigs. Based on this broad agreement we combined the seven libraries into a single analysis (Table 4). The top 30 piRNA clusters (i.e. clusters containing the largest number of unique potential piRNA sequences) were supported by all seven *Ae. aegypti *libraries in rough proportion to the sequencing size of each library. Furthermore, 77% or more of the sequences mapping to the top 30 piRNA clusters were found in only one of the seven *Ae. aegypti *libraries, suggesting that different sequences in different libraries supported the same piRNA clusters.

**Table 4:** Transposable element (TE) coverage and diversity in top *Ae. aegypti *piRNA clusters.

Cluster ID	Supercontig	start	stop	cluster TE coverage^1^	mean TE coverage of random sequences^2^	number of standard deviations, TE coverage^3^	number of TE elements in cluster^4^	mean number of TE elements in random sequences	number of standard deviations, number of elements
1	supercont1.478	490638	568444	0.226676263	0.419001465	-1.416991717	38	61.235	-1.304074578

2	supercont1.1	1151499	1278727	0.487436041	0.403024924	0.580732819	49	85.661	-1.343104567

3	supercont1.286	1260644	1444752	0.395222395	0.396091577	-0.005621894	64	107.978	-1.210704338

4	supercont1.943	196392	264127	0.736078304	0.416404001	2.293783988	44	54.717	-0.642122401

5	supercont1.209	235416	285783	0.410935515	0.419113904	-0.058733961	24	43.614	-1.499284524

6	supercont1.20	3110107	3224809	0.554179054	0.418089746	0.964456246	59	81.042	-0.944476926

7	supercont1.402	860224	953549	0.402781647	0.411314707	-0.059586613	42	69.042	-1.264686072

8	supercont1.1145	61909	94822	0.391930486	0.417567435	-0.178082295	22	30.692	-0.897834546

9	supercont1.435	926765	972082	0.302241935	0.419841185	-0.837861518	22	39.9	-1.508018889

10	supercont1.379	15854	108077	0.611099063	0.409120133	1.411986233	50	69.013	-0.893134363

11	supercont1.555	368190	374829	0	0.370578852	-1.834660452	0	7.416	-2.100509042

12	supercont1.209	305189	366980	0.459768255	0.423567623	0.271641456	22	51.899	-2.088008606

13	supercont1.109	91700	129810	0.51195193	0.426534479	0.603357822	17	35.09	-1.769148524

14	supercont1.697	504405	552871	0.498813626	0.428710684	0.485977742	30	42.633	-0.960185323

15	supercont1.164	1210657	1248495	0.572980258	0.42009945	1.044389637	35	34.491	0.047763239

16	supercont1.83	1415166	1433185	0.648834628	0.409050447	1.485209324	16	18.324	-0.361868136

17	supercont1.41	2610658	2644427	0.521261475	0.430863484	0.624645572	34	31.716	0.247718053

18	supercont1.2	275115	329629	0.446170779	0.422215779	0.174896759	53	46.562	0.478972045

19	supercont1.38	3097699	3148574	0.389653275	0.427616491	-0.271821406	52	43.976	0.62870919

20	supercont1.192	1700025	1763125	0.514270772	0.419413566	0.675173572	59	52.334	0.430866518

21	supercont1.38	2995043	3039699	0.538146315	0.428465514	0.764356465	40	39.557	0.037258641

22	supercont1.109	1912714	1927564	0.013803784	0.405080673	-2.303191497	5	15.475	-1.787236268

23	supercont1.194	895141	974760	0.510474755	0.421488238	0.642327725	64	62.082	0.1058288

24	supercont1.226	725419	748642	0.575051671	0.411773027	1.040816358	25	22.408	0.332107048

25	supercont1.518	151663	158105	0.458637281	0.360901428	0.487675463	14	7.232	1.906352115

26	supercont1.38	2572763	2684616	0.49881095	0.402104083	0.653081355	87	78.081	0.349121968

27	supercont1.90	907728	931721	0.420813537	0.422340266	-0.010037376	26	23.628	0.304378812

28	supercont1.2	1423334	1464722	0.508492595	0.4232601	0.598461412	35	37.239	-0.202802747

29	supercont1.192	551266	584318	0.405984328	0.414953558	-0.060505091	31	30.782	0.02131827

30	supercont1.1	1420282	1453931	0.61961367	0.423640435	1.324250019	26	31.445	-0.541018989

^1 ^Proportion the cluster with similarity to known *Ae. aegypti *TE sequences. Data derived from running the program RepeatMasker.

^2 ^1000 random sequences of the same bp. size as the cluster were used.

^3 ^Number of standard deviations between the the observed TE coverage and the mean TE coverage of random sequences

^4 ^Number of different kinds of TE sequences found in the cluster

The top 30 clusters ranged in size from 6 to 184 kb which a similar size range to those reported for *D. melanogaster *of 2 to 242 kb [6]. In *Ae. aegypti *the piRNA clusters occupied 20.6% of the assembled genome and could potentially generate 84% of the observed piRNAs. In comparison, *D. melanogaster *piRNA clusters have been reported to occupy only 3.5% of the genome and potentially produce 92% of the piRNAs [6]. The top 30 *Ae. aegypti *piRNA clusters were generally located either on different supercontigs or over 100 kb from each other on the same supercontig. The same analysis performed on our *D. melanogaster *library showed several piRNA clusters in closer physical proximity on the same chromosome (Table 3). This suggests that the *Ae. aegypti *piRNA clusters are more widespread and cover a greater proportional area of the genome than the clusters found in *D. melanogaster*. Many of the clusters, including the top 14, had piRNAs mapping predominantly to one strand. A similar bias was not observed when random genomic sequences of similar size to the clusters were examined (data not shown) suggesting that the observed bias was a characteristic of the individual clusters.

**Figure 5:**
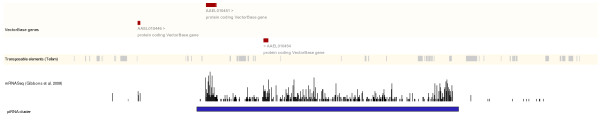
**The relative location of genes, transposons, and mRNA-seq sequences inside and surrounding the top *Ae. aegypti *piRNA cluster**. Location of the piRNA cluster is shown as a blue box near the bottom of the figure, the remaining genomic features were based on the output of the VectorBase *Ae. aegypti *genome browser for the region located on supercontig1.478:451736-607348.

piRNA clusters have been suggested as being possible regulatory loci of transposons and were reported in *D. melanogaster *to consist of 70-99% transposon sequences [6]. We did not observe such high proportions of transposon sequences in most *Ae. aegypti *clusters (Table 5). Comparison of *Ae. aegypti *piRNA clusters with random *Ae. aegypti *genomic sequences of similar size to the clusters did not reveal a statistically significant pattern of transposon density or diversity inside piRNA clusters. However, we did observe that in many clusters transposon sequences were all, or nearly all, in the same orientation. This has also been previously observed in some *D. melanogaster *piRNA clusters [6]. If our presumed piRNA clusters were the sites of piRNA biogenesis it might be expected they should show increased levels of transcription. Fortunately, the results of an *Ae. aegypti *mRNAseq analysis were available on the VectorBase *Ae. aegypti *genome browser (http://www.vectorbase.org) [48]. By superimposing cluster and mRNAseq information on the genome browser we observed that most of our piRNA cluster locations appeared to overlap with increased numbers of mRNAseq sequences (Figure 5). While in some clusters increased mRNAseq numbers were confined to our piRNA cluster boundaries, other clusters appeared to have high transcription levels both inside and in areas adjacent to the cluster that may be evidence we have underestimated the size of some of the piRNA clusters in *Ae. aegypti*.

**Table 5:** Number and percent abundance of piRNA and siRNA sequences in *Ae. aegypti *and *D. melanogaster *libraries, as well piRNA and siRNA sequences matching known TE sequences

	***Ae. aegypti***				***D. melanogaster***			
piRNA	number of piRNA sequences		percent of piRNAs		number of piRNA sequences		percent of piRNAs	
Total	11,173,973	(5,860,037)	100.00%	(100.00%)	271,626	(205,307)	100.00%	(100.00%)
TE	2,177,176	(1,240,908)	19.48%	(21.18%)	135,387	(110,068)	49.84%	(53.61%)
Retrotransposons	1,987,059	(1,114,018)	17.78%	(19.01%)	129,418	(105,485)	47.65%	(51.38%)

DNA transposons	113,030	(73,584)	1.01%	(1.26%)	5,969	(4,583)	2.20%	(2.23%)

MITE	29,558	(25,324)	0.26%	(0.43%)	N/A^2^	N/A	N/A	N/A

other^1^	47,529	(27,982)	0.43%	(0.48%)	N/A	N/A	N/A	N/A

**Endo-siRNA**	**number of siRNA sequences**		**percent of siRNAs**		**number of siRNA sequences**		**percent of siRNAs**	

Total	765,132	(446,834)	100.00%	(100.00%)	86,620	(54,049)	100.00%	(100.00%)

TE	216,859	(154,465)	28.34%	(34.57%)	15,870	(13,280)	18.32%	(24.57%)

Retrotransposons	133,042	(100,067)	17.39%	(22.39%)	15,276	(12,720)	17.64%	(23.53%)

DNA transposons	28,578	(18,692)	3.74%	(4.18%)	594	(560)	0.69%	(1.04%)

MITE	32,623	(23,667)	4.26%	(5.30%)	N/A	N/A	N/A	N/A

other^1^	22,616	(12,039)	2.96%	(2.69%)	N/A	N/A	N/A	N/A

Numbers in parentheses exclude duplicate sequences.

^1 ^TE elements such as Penelope that cannot be put into any of the other categories.

^2 ^Indicates that no elements from these categories have been reported in *D. melanogaster*.

Several *Ae. aegypti *piRNA clusters overlapped with annotated genes. Many, but not all, of these gene sequences contained large numbers of piRNA sequences. Interestingly, those genes that mapped to many piRNA sequences were nearly all oriented in the opposite orientation to the majority of piRNAs in the cluster. Cluster genes that did not contain many piRNA sequences showed no particular orientation bias for their piRNA sequences.

### Ae. aegypti piRNAs and endo-siRNAs generated to endogenous transposons

We combined the presumed piRNA sequences from all seven *Ae. aegypti *libraries into a single analysis (Table 5) and mapped these to annotated *Ae. aegypti *transposons in the RepBase and TEfam databases [49] (http://tefam.biochem.vt.edu/) (Additional File 2, Table S1). In our *D. melanogaster library*, almost 50% of presumptive unique piRNAs mapped to transposon sequences, slightly higher than the 42% seen for the 23-29 nt fraction obtained from *D. melanogaster *Oregon R total RNA [31]. In contrast, despite having a larger transposable element load than *D. melanogaster*, only 19.48% of the presumed piRNAs mapped to annotated transposable elements. It is entirely possible that this lower value may be in part due to unannoted transposable element sequences present in the *Ae. aegypti *reference genome. The majority (89.75%) of transposon-specific *Ae. aegypti *piRNAs mapped to class I transposons, 5.95% to class II transposons, 2.03% to MITEs and 2.27% to other transposons that currently cannot be easily assigned (Table 2 and Table S1). This reflects to some degree the relative abundance of the class I and class II transposons (without MITEs) in the *Ae. aegypti *reference genome (57% class I, 6.5% class II, 34.4% MITEs, 2.2% Helitrons [16]). Two LTR retrotransposons, Ty3_gypsy and *Pao_Bel *accounted for over 57% of all transposon-specific piRNAs with almost 44% mapping to the *Ty3_gypsy *retrotransposon (Additional File 2, Table S1). The number of unique piRNAs generated to MITEs is, however, proportionally less than their abundance in the genome. As described below, MITEs appear to be more preferentially targeted by endo-siRNAs and the relative lack of coding sequences may explain why proportionally few piRNAs map to them.

We generated piRNA density maps to seven *Ae. aegypti *transposable elements (Additional File 3, Figure S1). The class II transposon *AeBuster1 *is a member of the *hAT *superfamily and is active in interplasmid transposition assays performed in *Ae. aegypti *and *D. melanogaster *embryos [50]. Four intact copies are present in the genome reference strain. *AeTango2 *is also a class II transposon but belongs to the *IS630-Tc1-mariner *superfamily, is present in 25 copies in the *Ae. aegypti *genome of which one copy is possibly an active element based on bioinformatic properties (an intact transposase gene open reading frame, intact terminal inverted repeats, and the presence of the TA target site duplication) [51]. *Juan-A *(called jockey Ele1 in Tefam (http://tefam.biochem.vt.edu/)) is a non-LTR retrotransposon, is a member of the *jockey *clade, comprises approximately 3% of the entire genome sequence and is widely distributed amongst mosquito species in which it has been proposed to have been recently active [52]. *Lian *non-LTR retrotransposons (called LOA in Tefam) have been estimated to be present in the *Ae. aegypti *(Rockefeller) strain from approximately 460-1,380 copies per haploid genome based on the analysis of a genomic library. [53]. *MosquI*, (called I Ele1 in Tefam) is also a non-LTR retrotransposon, is a member of the *I *clade and, based on analysis of the same genomic library, is believed to be present in low copies [54]. *Pao_Bel-ele1 *and *Ty3_gypsy_ele1 *are both LTR retrotransposons with the former being present in 11 copies (four being full length) and the later being present in nine copies (four full length) [16] (http://tefam.biochem.vt.edu/). As described above, together these LTR retrotransposons account for almost 58% of the piRNAs that target transposons in the *Ae. aegypti *genome. All seven transposable elements were targeted by piRNAs and all but *MosquI-Aa2 *contained at least one ping-pong signature overlap within their transcripts implying that they could be subject to recognition and silencing by this pathway (Additional File 3, Figure S1). piRNAs mapped to both strands for all seven transposable elements but there was a strong anti-sense bias observed for the *AeTango2 *and *Ty3_gypsy_Ele1 *transposable elements and especially for the *Juan-A non-LTR *retrotransposon. These piRNA density maps of *Ae. aegypti *transposons are consistent with what has been found for *D. melanogaster *transposons [6,9,35]

In *D. melanogaster *endo-siRNAs have been shown to target transposons both in the germ line and the soma [10,55-57]. We investigated if this was also the case in *Ae. aegypti *by analyzing 21 nt long sequences in our libraries. In our *D. melanogaster *library 18% of sequences matched known transposons, a number slightly lower than a previous estimate of transposons in *D. melanogaster *cell cultures (27%, [55]) (Table 2). In the *Ae. aegypti *libraries 28% of sequences matched known transposons.

Because we used libraries derived from whole mosquitoes we were not able to distinguish between somatic and germ line-specific siRNA sequences. Nevertheless, it appeared likely that a large percentage of *Ae. aegypti *endo-siRNAs participate in interactions with transposons and could therefore be hypothesized to be at least partially involved in their regulation. Furthermore, it is interesting to note that a large number *Ae. aegypti *endo-siRNAs mapped to miniature inverted terminal element (MITE) sequences (Table 5). MITEs have, by definition, no coding potential and no such elements have been described from *D. melanogaster*. However, MITEs make up a substantial percentage (16%) of the assembled *Ae. aegypti *genome [16], but to date little is known about their transcription or regulation by the host genome. The substantially larger percentage of endo-siRNAs mapping to MITEs (15.03% of endo-siRNAs mapping to transposon sequences) than piRNAs (2.03%) may have its basis in their regulation or, more likely, arise from the production of foldback dsRNAs generated from the unidirectional transcription across the MITE terminal inverted repeats.

### piRNAs generated to introduced transposon sequences

**Figure 6:**
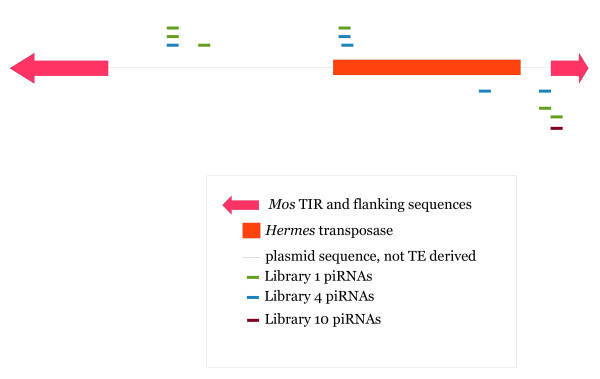
**Mapping locations of piRNAs to plasmid pMos3DB2Her that was used as a transformation vector for *Ae. aegypti *transformation**. Transposon derived sequences are shown as blocks. piRNA sequences are not drawn to scale to improve legibility. piRNA sequences shown above and below the plasmid sequence represent sequences that map to the positive and negative strand respectively, as determined by the transposase ORF. No piRNAs from libraries 2, 6, 11, or 12 mapped to this sequence.

The seven *Ae. aegypti *libraries included six libraries derived from mosquito lines that were germ line transformed with the *piggyBac*, *Mos1 *and *Hermes *transposons (Table 1). We attempted to detect the activation of a silencing mechanism targeted specifically at these introduced sequences by looking for small RNAs derived from them. We limited our analysis to piRNAs because siRNAs (21 nt) were considered too small to be reliably mapped for this analysis. In order to maximize the chances that any identified piRNA sequence had originated from the introduced sequences rather than from the host genome we ignored any piRNA sequence that mapped to the *Ae. aegypti *genome assembly and did not allow any mismatches between the piRNA and introduced sequences (Table 6, Figure 6). The analysis was slightly complicated by the fact that some of the transformation plasmids had regions of identical sequences, allowing piRNAs to appear to be derived from more than one plasmid. For example, library 1 was derived from a mosquito line transformed with the plasmid pMos3DB2Her; six piRNA sequences were found to match pMos3DB2Her, but also two piRNAs matched the plasmid autoHermes. However, these last two piRNAs were the same as we found to map to pMos3DB2Her and are therefore likely to be artifacts. Such likely artifact mapping events are indicated in Table 6 with parentheses.

**Table 6:** Number of piRNA and siRNA sequences that align to transposon sequences transformed into mosquito germlines.1

piRNA	1	4	2	6	11	12	10
template^2^	**pMos3DB2Her**		**pBac3EB2Mos**		**autohermes**	**pMos3DBhspPBac**	**Wild**

**pMos3DB2Her 3,175 (1,839) bp**.	**1(1)**	**7(4)**^3^	0	0	0	0	2(1)

**pBac3EB2Mos 3,520 (1,038) bp**.	0	0	**0**	**0**	0	0	0

**autohermes 3,081 (1,830) bp**.	0	0	0	0	**0**	0	0

**pMos3DBhspPBac 3,081 (1,785) bp**.	0	0	0	0	0	**0**	0
**siRNA**							
**pMos3DB2Her 3,175 (1,839) bp**.	**0**	**0**	0	0	0	0	0

**pBac3EB2Mos 3,520 (1,038) bp**.	0	0	**1(1)**	**1(1)**	0	0	0

**autohermes 3,081 (1,830) bp**.	0	0	0	0	**0**	0	0

**pMos3DBhspPBac 3,081 (1,785) bp**.	0	0	0	0	0	**0**	0

^1 ^All libraries but library #10 were derived from *Ae. aegypti *lines containing germline transformations of transposon sequences, the name of the transformation plasmid is indicated below each library number. Only matches to the transposon derived portions of the transformation plasmid are reported. Sequences that also match the *Ae. aegypti *genome assembly are excluded

^2^.Transposon sequence alignment template. Transposon sequences from these plasmids were used as templates for library alignments. Indicated next to each plasmid name is the size (bp) of the transposon derived sequences and in parentheses the size of the transposase sequence.

^3 ^Marked in bold are numbers where the mosquito line is transformed with the alignment template sequence. Numbers in parentheses indicate the number of alignments to the transposase.

**Figure 7:**
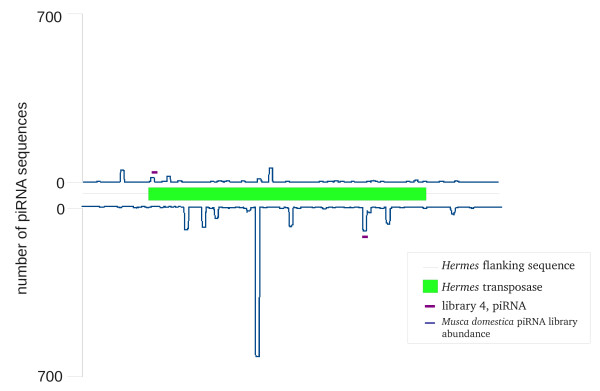
**Location of where two *Ae. aegypti *piRNA sequences from library 4 map to the *M. domestica Hermes *transposase**. Also shown is the abundance of piRNA sequences mapping to the sense and antisense strands of the *Hermes *transposon from a *M. domestica *small RNA library (abundance scales for both strands are shown on the left).

piRNAs were found to map both to transposon and non-transposon portions of introduced sequences (Figure 6). While the numbers were very small there appeared to be more piRNAs mapping to pMos3DB2Her in libraries 1 and 2 than in other libraries, suggesting that at least some of these piRNAs were indeed derived from the transformed sequence. We compared these to the presumptive piRNAs generated to *Hermes *from a small RNA library we constructed from its natural host, the housefly *Musca domestica *(Figure 7). While the number and types of piRNAs were far larger in housefly, two of these piRNAs were also generated in the transgenic *Ae. aegypti *line. piRNAs to *Hermes *were also generated to the transgenic lines containing an autonomous *Hermes *transposon (data not shown). This suggests that new piRNAs have been generated to introduced sequences sometime within the approximate 35 mosquito generations that separated the introduction of the transformation plasmids into the germ line and collection for library construction.

### piRNA generating genes

As described above, analysis of our *Ae. aegypti *libraries indicated that most transposon associated piRNA sequences were predominantly found within gene boundaries. Previous studies have described associations of individual genes with high numbers of piRNA sequences mapping to these genes [58-60]. We sought to identify such genes in *Ae. aegypti *by identifying genes sequences (defined here as including all annotated UTR, intron, and exon regions) with the highest density of uniquely mapping piRNAs. Because many *Ae. aegypti *genes have yet to be annotated with specific gene functions we attempted to assign a probable function to them by searching several databases (VectorBase, SwissProt, and NCBI) for similar genes with known functions (Table 7).

**Table 7:** The top 30 *Ae. aegypti *genes with highest piRNA density

Gene	Gene length (bps.)	Unique mapping piRNAs^1^	piRNA density (piRNAs/gene bps.)	strand percent +/-	gene inside piRNA cluster	Function of best match (if available) and species	Best match database	Best match accession
AAEL011224	303	16488	54.6	100/0	yes	hypothetical protein	VectorBase	AAEL011224

AAEL007866	1334	10651	8.0	99/1	yes	putative NS1 protein, *Aedes flavivirus *	NR	YP_003084126

AAEL007861	1182	4356	3.7	100/0	yes	uncharacterized transmembrane protein DDB_G0289901, *Dictyostelium discoideum*	Swiss-Prot	Y8625_DICDI

AAEL010454	1425	4084	2.9	90/10	yes	hypothetical protein	VectorBase	AAEL010454

AAEL000120	1020	1432	1.4	94/6	no	nucleoprotein N, bovine ephemeral fever virus	Swiss-Prot	NCAP_BEFV

AAEL009005	629	856	1.4	100/0	no	adult cuticle protein, *Aedes aegypti*	VectorBase	AAEL009005

AAEL005277	4038	4891	1.2	100/0	yes	Zinc finger protein on ecdysone puffs, *Drosophila melanogaster*	Swiss-Prot	PEP_DROME

AAEL009525	1236	1494	1.2	100/0	no	nucleoprotein putative, *Aedes aegypti*	VectorBase	AAEL009525

AAEL005213	1377	1582	1.1	100/0	no	hypothetical protein	VectorBase	AAEL005213

AAEL000668	1333	1522	1.1	100/0	no	conserved hypothetical protein	VectorBase	AAEL000668

AAEL001004	6251	7009	1.1	100/0	no	protein maelstrom homolog, *Aedes aegypti*	Swiss-Prot	MAEL_AEDAE

AAEL007686	2253	2439	1.1	100/0	no	Transcription factor MafB, *Coturnix coturnix jopnica *	Swiss-Prot	MAFB_COTJA

AAEL001772	2562	2643	1.0	100/0	no	RNA-dependent RNA polymerase, Nyamanini virus	NR	YP_002905337

AAEL005456	966	841	0.9	100/0	yes	nucleoprotein putative, *Aedes aegypti*	VectorBase	AAEL005456

AAEL000808	978	811	0.8	100/0	yes	nucleoprotein putative, *Aedes aegypti*	VectorBase	AAEL000808

AAEL013013	1088	862	0.8	100/0	no	hypothetical protein	VectorBase	AAEL013013

AAEL006843	21911	17268	0.8	100/0	no	NEDD4-binding protein 2, *Homo sapiens*	Swiss-Prot	N4BP2_HUMAN

AAEL006159	330	247	0.8	100/0	yes	Peritrophin-1, *Anopheles gambiae*	Swiss-Prot	PE1_ANOGA

AAEL005225	762	570	0.7	100/0	no	conserved hypothetical protein	VectorBase	AAEL005225

AAEL004959	1242	914	0.7	100/0	no	nucleoprotein putative, *Aedes aegypti*	VectorBase	AAEL004959

AAEL009873	1602	1142	0.7	78/22	no	nucleoprotein, bovine ephemeral fever virus	Swiss-Prot	NCAP_BEFV

AAEL005768	738	511	0.7	100/0	no	hypothetical protein	VectorBase	AAEL005768

AAEL007499	579	372	0.6	100/0	no	hypothetical protein	VectorBase	AAEL007499

AAEL007844	2572	1490	0.6	100/0	no	glycoprotein, spring viremia of carp virus	NR	ABB13496

AAEL010887	60098	34174	0.6	100/0	no	Myosin heavy chain IB, *Acanthamoeba castellanii*	Swiss-Prot	MYSB_ACACA

AAEL000673	1461	753	0.5	100/0	no	arbohydrate kinase domain-containing protein, *Mus musculus*	Swiss-Prot	CARKD_MOUSE

AAEL000276	71446	36638	0.5	1/99	no	VPRBP protein, *Culex quinquefasciatus*	NR	XP_001865214

AAEL012346	1177	598	0.5	100/0	no	hypothetical protein	VectorBase	AAEL012346

AAEL006704	1356	684	0.5	100/0	no	fibrinogen and fibronectin, *Aedes aegypti*	VectorBase	AAEL006704

AAEL003715	615	298	0.5	100/0	no	hypothetical protein	VectorBase	AAEL003715

**Figure 8:**
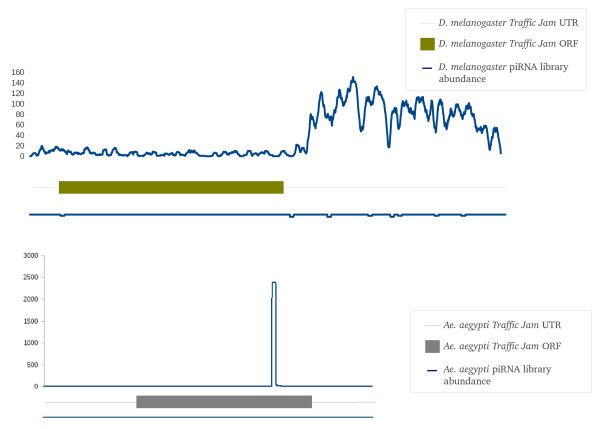
**The abundance of *D. melanogaster *piRNA sequences mapping to the sense and antisense strands of the *D. melanogaster tj *gene (top) and *Ae. aegypti *piRNA sequences (all libraries combined) mapping to the *Ae. aegypti *homolog of the *tj *gene (bottom)**. Abundance scale is shown on the left.

**Figure 9:**
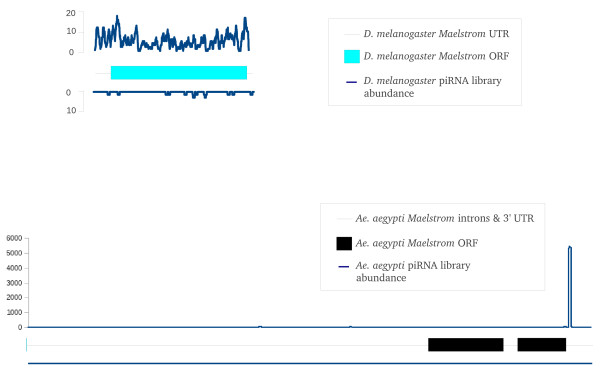
**The abundance of *D. melanogaster *piRNA sequences mapping to sense and antisense strands of the *D. melanogaster maelstrom *gene (top) and *Ae. aegypti *piRNA sequences (all libraries combined) mapping to the *Ae. aegypti *homolog of the *maelstrom *gene (bottom)**. Abundance scale is shown on the left.

While this is a preliminary overview of the piRNA-gene association in *Ae. aegypti*, several features stand out. Among the top 30 genes eight have strong similarities to viral-derived sequences with all of them having the majority of their piRNAs mapping to the antisense strand. While several previous studies in mosquitoes have linked silencing of viral transcripts with 21 nt viRNAs, a definitive link between piRNAs and viral sequences in mosquitoes has yet to be well established [61-63]. Two *Ae. aegypti *genes in Table 7 have similarities to genes previously associated with possible transposon regulation. Gene AAEL001004 appears to be an *Ae. aegypti *homolog of the *D. melanogaster maelstrom *gene. *Maelstrom *localizes to the nuage and has been implicated in transposon regulation in mice and *D. melanogaster *[64-66]. The second gene of interest is AAEL007686 that has sequence similarities with the MafB transcription factor and is a homolog of the *D. melanogaster traffic jam *gene. *Traffic jam *has been described as a piRNA cluster, with most of the piRNAs arising from the sense strand of its 3' UTR [60]. While the *Ae. aegypti *gene AAEL007686 could possibly give rise to at least 2,439 piRNAs (Table 7) it was not found inside an identified piRNA cluster. The great majority of piRNAs mapped to the sense strand of AAEL007686 but, in contrast to *D. melanogaster traffic jam*, piRNAs mapped predominantly to the 3' end of the open reading frame rather than to the UTR (Figure 8). 98.3% of piRNAs mapping to the *Ae. aegypti tj *gene contain U in their first position while 75.3% commenced with the 25 nt sequence 5' UAUUGACAACAGAAGUAACGAAUGA 3' with most variations being in the small number of additional ribonucleotides present at the 3' end. We confirmed using 3'RACE that the actual transcription termination site of this gene was consistent with its annotation and also confirmed, using RNAseq data, that this piRNA site was located in the translated part of the transcript (Additional File 4, Figure S1). We also examined the piRNA density of the *D. melanogaster tj *gene using our own *D. melanogaster *library and published *D. melanogaster *piRNA libraries [6,46] and confirmed the previous location of the majority of these to the sense strand of the 3' UTR (Figure 8)[60]. Interestingly, the *Ae. aegypti maelstrom *piRNAs all map to the 3' UTR of the sense strand, which we also confirmed by RNAseq analysis of this gene (Figure 9, Additional File 5, Figure S1). 88.6% of these piRNAs contained A at the 10^th ^position of the piRNA. We examined our own and published *D. melanogaster *libraries [6,46] for piRNAs mapping to *mael *and found low numbers of them throughout the transcript, mapping mainly to the sense strand (Figure 9).

## Discussion

High-throughput sequencing technology has greatly increased the opportunities to study the possible regulatory roles of piRNA and siRNA sequences. In arthropods most of the research has focused on the piRNA sequences of *D. melanogaster *(reviewed in [13,58]), where it has emerged that a major role of piRNAs is the regulation of transposons. However, *D. melanogaster *has a relatively small percentage of its genome composed of transposon derived sequences and has no described MITEs which are present in many of other insect genomes (including other drosophilids). We have conducted an analysis of piRNAs in the mosquito *Ae. aegypti*, which is a vector of many arboviruses and contains a genome rich in transposon derived sequences (47%) which includes a significant percentage of MITEs derived sequences (16%) [16].

Sequencing of seven whole tissue libraries sampled only a portion of all available piRNA sequences in this mosquito. Based on sequence overlaps between these libraries we estimated that the total diversity of piRNA sequences in *Ae. aegypti *was within the same order of magnitude as the piRNA diversity of *D. melanogaster*. Given this similarity in overall piRNA diversity between *Ae. aegypti *and *D. melanogaster *it was surprising that only 19% of *Ae. aegypti *piRNAs mapped to annotated transposon sequences, compared to 50% of piRNAs in our *D. melanogaster *library. Barring a large number of unrecognized transposons in *Ae. aegypti*, this suggests that the role of piRNAs in transposon regulation in *Ae. aegypti *follows the *D. melanogaster *model in some, but not all, respects.

The majority of the 19% of piRNAs that did map to transposons in *Ae. aegypti *mapped to the antisense transcription strand, a pattern that was previously observed in *D. melanogaster*, and is consistent with having transposon transcripts sliced by PIWI proteins loaded with piRNAs that are antisense to the transposon transcript [31]. *Ae. aegypti *piRNA sequences mapped to all transposon classes including MITEs (Table 2). MITEs may be transcribed but do not produce a functional protein product and so, in order to be mobilized require the presence of a corresponding full length and active transposase [67]. *Ae. aegypti *MITEs show little sequence similarity to full length transposons making it unlikely that many of the observed piRNAs mapping to MITEs are simply remnants of full length transposon inactivation systems. However, only 0.26% of *Ae. aegypti *piRNAs mapped to MITEs despite them comprising 16% of the assembled genome. Conversely 4% of *Ae. aegypti *endo-siRNAs mapped to MITEs which is perhaps not surprising given that transcription along the length of a MITE would produce a foldback dsRNA sequence that would elicit the siRNA response. A link between transposons, miRNAs and gene regulation was first proposed from studies in humans and has been refined, based on analyses performed in plant genomes, to include both siRNAs and MITEs [68-71].

We were able to identify a number of piRNA clusters that generated a large percentage (84%) of observed piRNAs. A number of lines of evidence supported these genomic locations as having a similar role to *D. melanogaster *clusters. First, previously described *D. melanogaster *clusters were also recovered using our cluster discovery procedure applied to our *D. melanogaster *library (Table 3). Second, all *Ae. aegypti *libraries supported the same basic piRNA cluster locations (Table 4). Third, the top 30 *Ae. aegypti *clusters appeared to overlap with transcribed portions of the genome (Figure 5). Finally, these clusters shared some common features with previously described *D. melanogaster *piRNA clusters. These included: roughly similar ranges in individual cluster lengths; a mixture of clusters with piRNAs mapping almost exclusively to one strand and clusters with piRNAs mapping to both strands; and similarities in the relative orientation of piRNA, transposon, and transcription orientation for at lease some clusters. Some piRNA clusters in *D. melanogaster *and mouse are transcribed unidirectionally. This is the case for the *D. melanogaster flamenco *locus in which 99% of piRNAs map to the sense strand of transcription, while all the transposon sequences are oriented in the direction opposite to transcription [31]. Many of the top *Ae. aegypti *piRNA clusters also contained transposon sequences predominantly oriented in the same direction (Table 5). Unfortunately, overall cluster transcription direction could not be determined for all clusters. However, among the 30 piRNA clusters in Table 4 three overlapped with protein coding genes that were also identified as generating piRNAs (cluster 1, gene AAEL010454; cluster 3, genes AAEL007861 and AAEL007866; cluster 29, gene AAEL006159; Tables 4 and 7). If we assume that the clusters are transcribed in the same orientation as these genes we see a similar pattern in clusters 1 and 3 to the *D. melanogaster flamenco *locus: piRNAs are on the sense strand of transcription while transposon sequences are oriented in the opposite direction to transcription (in cluster 29 transposon were not predominantly oriented in any one direction). These observations further reinforce the similarity between *Ae. aegypti *and *D. melanogaster *piRNA clusters. However, while piRNA clusters in *D. melanogaster *and mouse generate sequences that predominantly map to transposons [4,6] fewer than a quarter of potential piRNAs generated from *Ae. aegypti *clusters matched known transposons. Furthermore, while many previously described piRNA clusters contained a high density of transposon sequences we did not detect significantly higher levels of transposon sequences within *Ae. aegypti *clusters compared to random portions of the genome (Table 5). However *Ae. aegypti *piRNA clusters covered a greater portion of the assembled genome than *D. melanogaster *clusters and so may be more widespread. These results suggest that while *Ae. aegypti *and *D. melanogaster *share many features of their piRNA clusters, the role these clusters have in transposon inactivation may not be completely identical between these species. The nature of this difference has yet to be determined.

We examined the piRNA density maps to seven *Ae. aegypti *transposons and found these to be similar in their patterns to equivalent density maps from *D. melanogaster *[6,9,35]. All but the non-LTR *MosquI *element contained at least on ping-pong amplification overlap suggesting that these could be silenced by this pathway. Notably these ping-pong signatures were present in representatives of the two LTR elements that together account for 58% of piRNAs. The most marked anti-sense strand bias was observed for the non-LTR *JuanA *element which has been proposed to be recently active in mosquitoes [52]

To better understand the role of the 81% of *Ae. aegypti *piRNAs that did not map to transposons we examined possible associations between protein coding genes and piRNA sequences. In addition to their role in transposon transcript degradation, piRNA sequences have been demonstrated to silence protein coding genes in *D. melanogaster *[60,72]. The *Supressor of Stellate *(*Su(ste)*) and *Traffic Jam *(*tj*) genes contain piRNA sequences on their sense strand that can be used to degrade the transcripts of the *Stellate *(*Ste*) and *Fasciclin 3 *(*Fas 3*) gene transcripts respectively [60,72]. *Ae. aegypti *contained a number of genes with piRNAs mapping almost exclusively to their sense strand that are therefore unlikely to be involved in the regulation of the host gene transcript (Table 7). Instead, they may be used to regulate other genes, the identity of which cannot be deduced using the present data. However, it is interesting to note that just as the *D. melanogaster tj *gene has been identified as a source of piRNAs, a possible ortholog of *tj *in *Ae. aegypti *may also be a source of piRNA sequences (AAEL007686, Table 7). Furthermore, a putative ortholog to the *D. melanogaster Fas 3 *gene has been identified in *Ae. aegypti *(AAEL003044, OrthoDB http://cegg.unige.ch/orthodb4) suggesting that the *Ae. aegypti *AAEL007686 gene has at least the potential to act in a similar way to the *D. melanogaster tj *gene. One difference in the location of the sense-strand piRNAs arising from both *Ae. aegypti *and *D. melanogaster *is that for *Ae. aegypti tj *they are located upstream from the translation termination codon rather than being located within the 3' UTR.

The location of piRNAs to the sense strand of the 3' UTR of the *Ae. aegypti maelstrom *(*mael*) gene suggests that these piRNAs may also be involved in the regulation of downstream genes, as had been proposed for *D. melanogaster tj *[60] However as yet we are unable to identify these target genes. In *D. melanogaster*, *mael *is associated with both the nucleus and nuage of germ line cells [64]. Mutations in *Mael*, as well as in other components of the nuage, such as *vasa *and *Krimper*, have been shown to reduce the levels of piRNAs associated with the *HeT-A*, *roo *and *I *transposons suggesting that these genes play a role in the suppression of transposition in the *D. melanogaster *female germ line [66]. Consistent with this is the role of *Mael *in mouse spermatogenesis in which its absence led to a 100-fold increase in L1 expression and a 3-5 fold increase in the expression of the unrelated IAP element [73]. As was seen for both *Ae, aegypti *and *D. melanogaster tj*, the piRNAs map to the sense strand of *mael *but differ from *Ae. aegypti tj *in that they are located in the 3' UTR. We find it encouraging that two of the top 30 piRNA generating *Ae. aegypti *genes have previously implicated in the regulation of either piRNAs or transposons in *D. melanogaster *and so suggests that our own bioinformatics screening of these libraries is generating valid targets. Seven other protein coding genes also generated piRNAs only to the sense strand and all remain unannotated (AAEL011224, AAEL017228, AAEL005277, AAEL005213, AAEL009263, AAEL013013, AAEL011027)(Table 7). It remains to be determined if piRNAs from many of these genes arise from the exonic sequence since many current *Ae. aegypti *gene annotations have not been manually curated and are based mostly on relatively poor EST datasets. Robine *et al*. (2009) noted that in mouse piRNA libraries many piRNA clusters that were once believed to be exonic could, upon reexamination, be reclassified as 3'UTR directed. This same phenomenon of high density of piRNAs on the sense strand of the 3' end of the ORF was also observed in the case of the putative *maelstrom *homolog gene described above.

*Ae. aegypti *is a vector of many RNA viruses, some of which cause severe disease in humans. Eight of the 30 top piRNA generating genes in *Ae. aegypti *are apparently of viral origin (Table 7). Three of these generate piRNAs only to the antisense strand (AAEL007866, AAEL017001, AAEL007844,) while another (AAEL009873) generates 99% of it's piRNA to this strand. The remaining three (AAEL017355, AAEL000120, AAEL001772) generate piRNAs mapping to both strands, although for each it is the antisense strand that dominates. This may indicate that most of the piRNAs generated to viral-like genes function by, in association with the appropriate Piwi protein, slicing the viral gene transcripts. As such this mechanism is entirely different to that operating for *tj*, *mael *and the seven other protein coding genes in which the piRNAs are generated exclusively by the sense strand. All of the remaining 12 protein coding genes that generate piRNAs have these mapping primarily to the antisense strand.

There has been some recent evidence implicating piRNAs in the recognition of arboviruses in *Ae. aegypti *and *Aedes albopictus*. In addition to siRNAs, small RNAs 24-30 nt in length to the sense strand of the dengue virus genome were recovered from infected *Ae. aegypti *and none showed a bias for uracil at the 5' end and little bias for adenine at the 10^th ^position although these authors stated that unpublished data revealed that small RNAs of the same size distribution generated to the sense strand of Sindbis virus did show a U1 bias [63]. *Aedes albopictus *C6/36 cells have been found to lack an siRNA response to infection by West Nile virus, Sindbis virus and La Crosse virus, but do generate small peaks of smaller RNAs 24-28 nt in size to Sindbis and La Crosse virus infections yet no such peak is generated by infection with the dengue virus [62,74]. Interestingly small RNAs within this size range generated to the inadvertent infection of C6/36 cells by Cell Fusing Agent virus showed a strong preference for adenine at the 10^th ^position which is consistent with them being piRNAs that interact with, in D. melanogaster, the AGO3 protein [74]. Taken together these data from two difference *Aedes *species indicate that a piRNA response to arboviral infection may be generated and, if so, implicate this pathway in an anti-viral response. Taken in this context, the piRNAs generated by the viral-like sequences identified here may be further evidence of the role that this small RNA pathway may play in anti-viral defense in this mosquito. *Ae. aegypti *may thus provide important and novel information concerning how this small RNA pathway interacts with both transposons and viruses, both of which are abundant in this insect, especially in comparison with *D. melanogaster*.

A large portion of transposon-matching *Ae. aegypti *piRNAs mapped inside genic sequences for reasons that remain unclear (Figure 1A). Some protein coding genes are likely origins of piRNAs (Table 7), but it appears unlikely that these would be sufficient in number to account for this observation. A possible, but as yet untested, explanation might be that these regions contain a high level of active transposons. Genic regions of the genome are more likely to be transcribed, which may increase the chances that an inserted transposon will actively transpose. This in turn could produce a higher response of piRNA silencing mechanism to these transposons.

As a vector of human disease pathogens there is interest in developing highly robust genetic tools for *Ae. aegypti*. While germ line transformation is possible (albeit a low rate compared to *D. melanogaster*) efforts to remobilize transposons in *Ae. aegypti *have occurred at very low rate suggesting the presence of a transposon silencing mechanism [21,23,41,75]. We examined the piRNA content of mosquito lines that had been transformed with transposon sequences and found preliminary evidence that piRNA sequences mapping exclusively to the transformed sequences had been produced. Since these piRNAs did not match the current *Ae. aegypti *assembly their presence in the transformed lines was likely explained in one of two ways: 1) they had been maternally inherited and perhaps amplified via the ping-pong cycle, or 2) new piRNAs were being generated from introduced sequence. In either case these data are suggestive that a component of the piRNA pathway was activated by the insertion of foreign DNA into the genome although we have no information as to how rapid this response would have occurred. The full kinetics of the piRNA response to transgenic sequences need to be explored in association with genome-wide transcriptional analyses which should shed light on the relationship between transgenesis, the small RNA response and viral infection in this mosquito

## Conclusions

We analyzed piRNA and endo-siRNA sequences from *Ae. aegypti*, a mosquito that is a significant vector of human pathogens and has a large genome size with a correspondingly high transposon content. Unlike *D. melanogaster*, *Ae. aegypti *contains MITEs and we found higher levels of siRNAs targeted to these than piRNAs. The terminal inverted repeats of MITEs most likely enables foldback RNAs to be formed from unidirectional transcripts, leading to the induction of the siRNA pathway, which is associated more with anti-viral defense. Despite having an abundance of transposons, the majority of piRNAs in *Ae. aegypti *were targeted to non-transposon sequences, many of which were protein-coding genes. As such the piRNA profile of this mosquito is more similar to that of mice than *D. melanogaster *in which the majority of piRNA sequences map to transposons. The majority of piRNAs in this mosquito were 28 nt in length and so longer than those seen in *D. melanogaster *but contained the U1 or A10 sequence bias seen in other organisms in which piRNAs have been sequenced. Two genes targeted by piRNAs in *Ae. aegypti *have been implicated in piRNA biogenesis or function while the function of the majority of them remain unknown. Several others were of viral origin suggesting that the piRNA response may extend into anti-viral defense in this insect. piRNAs were also generated to introduced transposons. The diversity of endogenous transposons present in this mosquito, together with the corresponding diversity and number of piRNAs and siRNAs mapping to them suggests that these small RNA pathways may of some importance in maintaining the integrity of its genome in the presence of numerous transposons and viruses.

## Methods

### Purification of Small RNAs from *Ae. aegypti *and *D. melanogaster*/Library Construction

Total RNA was extracted from approximately 200 mosquitoes using Trizol reagent (Invitrogen). 10-20µg of the total RNA was run on a 15% polyacrylamide/7M urea/TBE gel using a Hoeffer SE420 electrophoresis apparatus (Hoeffer). Gel bands corresponding to approximately 16 to 35 bases were excised. The Illumina small RNA sample prep kit (Illumina) was used for all steps of library construction. Gel bands were broken up by centrifugation through small holes in 0.5 ml microfuge tubes and RNA was eluted with 0.3M NaCl. Following gel removal with Spin-X filters and precipitation with glycogen and ethanol, samples were resuspended in water. Small RNAs were ligated to the SRA 5' adapter overnight before size selection on a 15% polyacrylamide/7M urea/TBE gel. Gel bands corresponding to approximately 40-60 bases were excised and purified as above. The samples were next ligated overnight to the 3' adapter before purification on a 10% polyacrylamide/7M urea/TBE gel. Gel bands corresponding to 70-90 bases were excised and purified. Reverse transcription was performed using Superscript III (Invitrogen) before library amplification with Phusion DNA polymerase. Amplification was as follows: 98ºC for 30 seconds, followed by 15 cycles of: 98ºC 10 seconds, 60ºC for 30 seconds, and 72ºC for 15 seconds, with a final step of 10 minutes at 72ºC. The final library was purified by size selection of gel bands corresponding to 85-110 bp on a 6% polyacrylamide/TBE gel. Library quality was assessed by ligation into the pJET1.2 vector (CloneJet kit, Fermentas) followed by standard DNA sequencing. Final library sequencing was performed by the staff of the UCR Institute for Integrative Genome Biology on the Illumina GAx2 sequencer.

### Processing libraries

Sequences were bioinformatically stripped of adapters using R scripts. Following this ribosomal sequences were removed by mapping each library to a database containing all known ribosomal RNAs (rRNA, tRNA, snRNA, etc.) derived from Genbank records (http://www.ncbi.nlm.nih.gov/genbank/) for the appropriate genome and removing any sequences with significant matches. A similar process was used to remove miRNAs using the sequences deposited in mirBase (http://www.mirbase.org/) to identify *Ae. aegypti *and *D. melanogaster *miRNAs. Finally, sequences were mapped (see below) either to the *Ae. aegypti *assembly available at VectorBase (http://www.vectorbase.org) or to the BDGP Release 5 *D. melanogaster *assembly (http://www.fruitfly.org/). All analyses were limited to sequences that mapped to the reference genome with the exception of the analysis of piRNAs mapping to transformed sequences, where all piRNAs were used.

### Mapping sequences to genomes and other databases

Mapping was mostly performed using the program Bowtie [76]. Mapping to the genome was performed using a seed length of 30 bp and allowing up to 2 mismatches within the seed. Mapping to other databases did not use a seed, but instead required a match along the entire length of the sequence with up to 2 bp mismatches. The only mapping not performed using Bowtie, was matching the sequenced libraries to ribosomal databases (see above) which was performed using the BLAT program with the "-fastMap" option [77].

### Estimating piRNA abundance

We based our estimates of the size of the piRNA sequence pool in *Ae. aegypti *and *D. melanogaster *on the observed number of piRNAs in each library (i.e. sequences 24 nt long or larger) and on the amount of overlap between libraries. These were used with the formula described previously [45]. However, based on simulation experiments we designed to verify this method *in-silico *it appeared that including highly duplicated sequences into the calculation could have a large negative impact on the estimates. We minimized this effect by excluding from the analysis any sequence that was duplicated in any one library. We estimated the size of *Ae. aegypti *piRNA pool using every possible pair of *Ae. aegypti *libraries that were not replicates (19 estimates) and averaged these for a final estimate of the piRNA pool size. For *D. melanogaster *we used eight published libraries [46] deposited at the National Center for Bioinformatic Infomation GEO database under record GSE30955. Only sequences that were 24 nt long or larger and that mapped to *D. melanogaster *genome assembly were used from these eight libraries to minimize the chances that non-piRNA sequences were included. Estimates were performed on all possible pairs of the eight libraries (28 estimates).

### Periodate oxidation and β-elimination of small RNAs

For analysis of the chemical structure of the 3' ends of small RNAs from *Ae. aegypti*, total RNA was purified using Trizol reagent (Invitrogen) from 2 day post- blood-fed females. RNAs of approximately 28-32 nt were purified from 10 *µ*g total RNA on a 15% polyacrylamide gel containing 7.5 M urea. RNA ladder was obtained from Illumina, and the control 23-mer synthetic RNA was the kind gift of Dr. Shou-wei Ding (University of California, Riverside). Following removal of 5'-phophates with FastAP (Fermentas), RNAs were labeled with ^32^P-ϒ-ATP and T4 polynucleotide kinase (Fermentas). The method for the β-elimination was according to published protocols [32]. Signals were visualized with BioMax film (Kodak).

## Authors' contributions

PA carried out bioinformatics analyses, RHH performed molecular genetic studies and library constructions, JAW participated in molecular genetic analyses, library constructions, and bioinformatic analysis, NLC participated in experimental design and edited the manuscript, PWA conceived of the study, developed the experimental design and wrote the manuscript with editorial assistance from all co-authors who approved the final manuscript.

## Supplementary Material

Additional file 1 **Figure S1**. Size distribution of *Ae. aegypti *small RNA abundance in each of the seven *Ae. aegypti *libraries and the single *D. melanogaster *library. The number of small RNAs mapping to *Ae. aegypti *genes, transposons, both, or neither, are shown as different colors for each size class (the legend is shown on the right). Click here for file

Additional file 2 **Table S1**. Number of piRNAs from *Ae. aegypti *libraries mapping to Transposable Element (TE) family consensus sequences and percentage occupancy of the genome by TE families. Click here for file

Additional file 3 **Figure S1**. Small RNA (>= 24 nt.) density plots for representative *Ae. aegypti *full length transposable elements. Small RNA density for all sequenced *Ae. aegypti *libraries mapping to the sense strand of the transposable element is show in red, mapping to the anti-sense strand shown in blue. Position and density of possible U1A10 overlap pairs is shown in light blue. Position of the ORF(s) is shown at the bottom of each figure. Click here for file

Additional file 4 **Figure S1**. piRNA density, mRNA-seq transcript coverage and assembly of the *Ae. aegypti *genomic region surrounding a putative *Ae. aegypti *homolog of the *traffic jam *gene. Genomic region supercontig identity and boundaries are shown below the piRNA density graph. mRNA-seq data were derived from an *Ae. aegypti *ovary tissue library. mRNA-seq transcript assembly, shown in blue, was performed using the CUFFLINKS program (Trapnell et al. 2010). Location on the genomic region of the mRNA transcript assembly and gene annotation, as reported in VectorBase, are show at the bottom. Click here for file

Additional file 5 **Figure S1**. piRNA density, mRNA-seq transcript coverage and assembly of the *Ae. aegypti *genomic region surrounding a putative *Ae. aegypti *homolog of the MAELSTROM gene. Genomic region supercontig identity and boundaries are shown below the piRNA density graph. mRNA-seq data were derived from an *Ae. aegypti *ovary tissue library. mRNA-seq transcript assembly, shown in blue, was performed using the CUFFLINKS program (Trapnell et al. 2010). Location on the genomic region of the mRNA transcript assembly and gene annotations, as reported in VectorBase, are show at the bottom. Reference cited in Additional file 5, Figure S1. 1. Trapnell et al., "Transcript assembly and quantification by RNA-Seq reveals unannotated transcripts and isoform switching during cell differentiation," *Nature Biotechnology *28, no. 5 (2010): 511-515. Click here for file
