# Supplementary figures and images for: The mosquito Aedes aegypti has a large genome size and high transposable element load but contains a low proportion of transposon-specific piRNAs
Source: BMC Genomics. 2011 Dec 15;12:606. doi: 10.1186/1471-2164-12-606 (PMC3259105; doi:10.1186/1471-2164-12-606)

number of uniquely mapping sequences

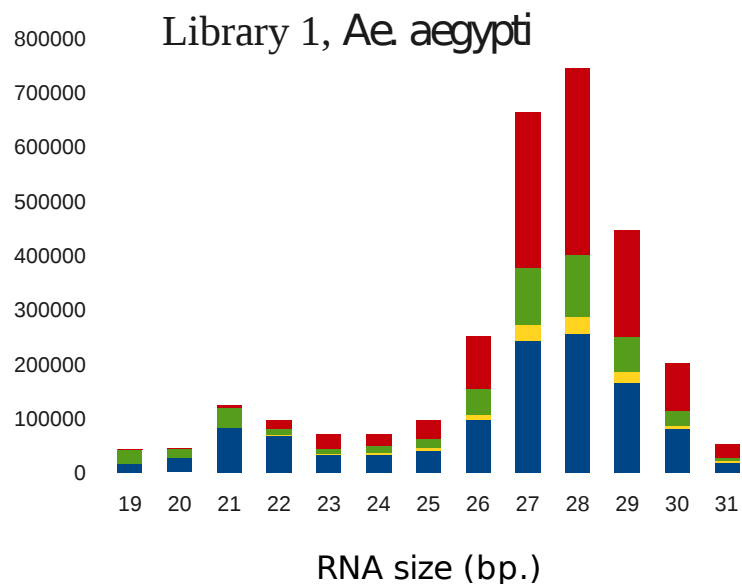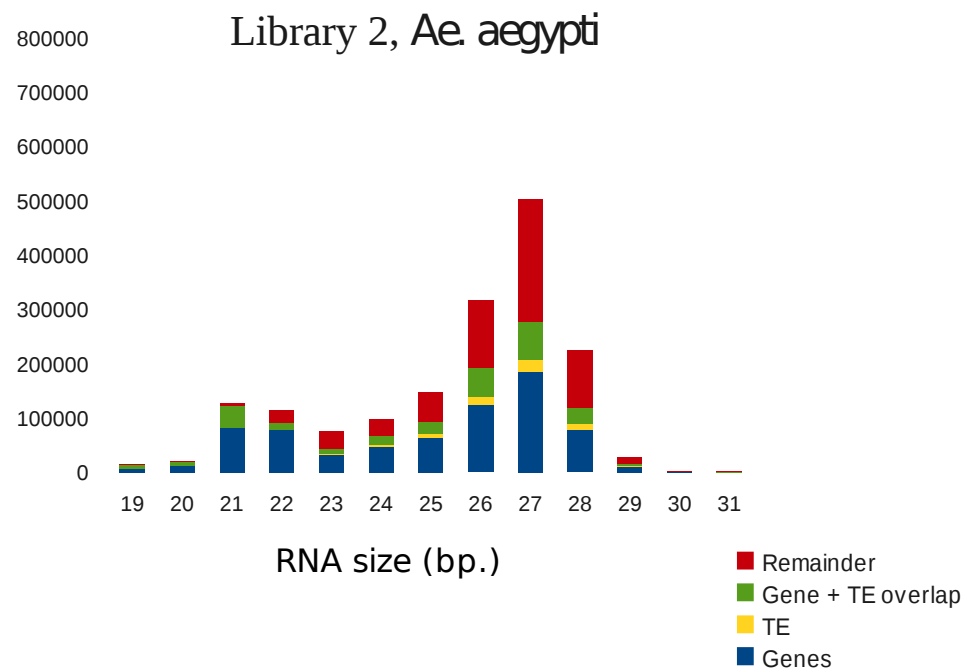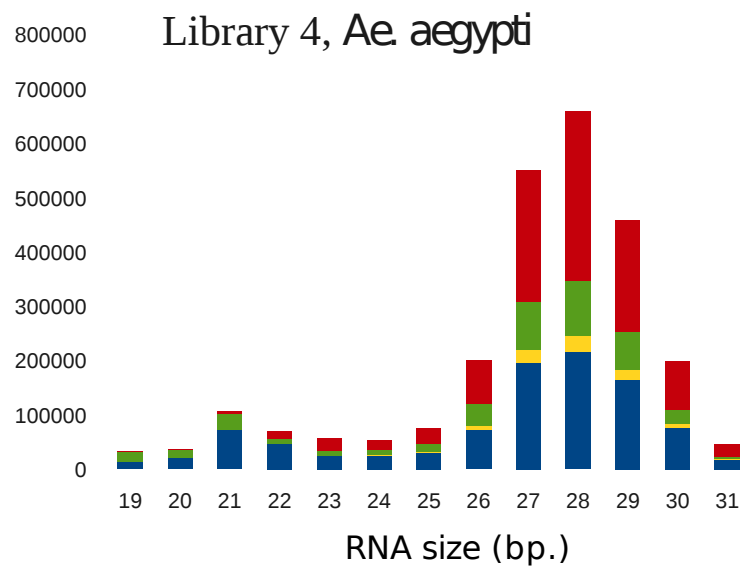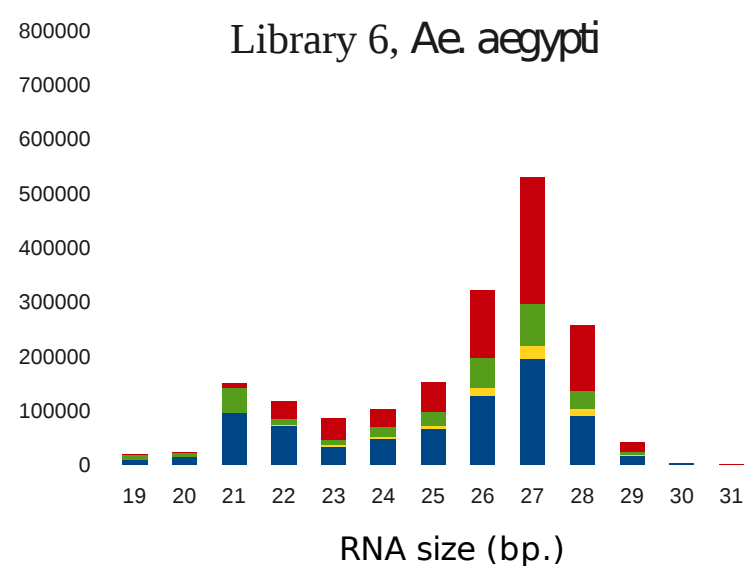

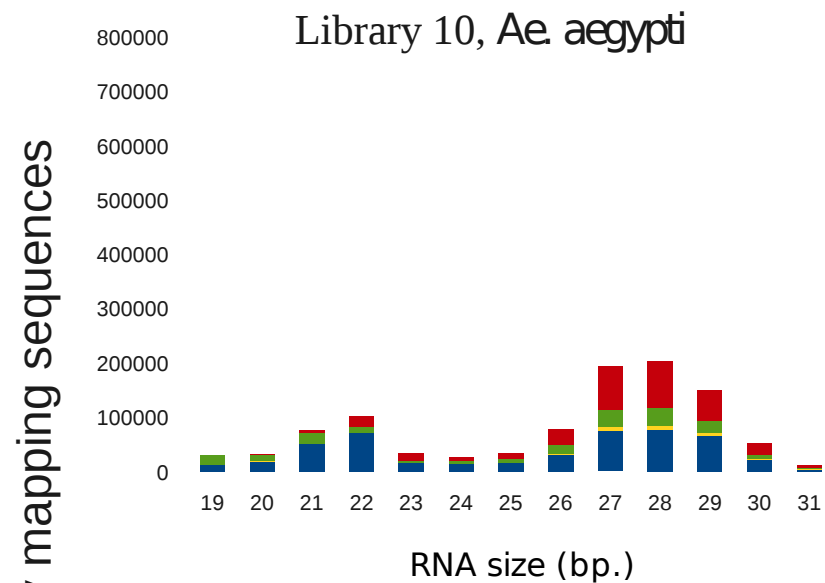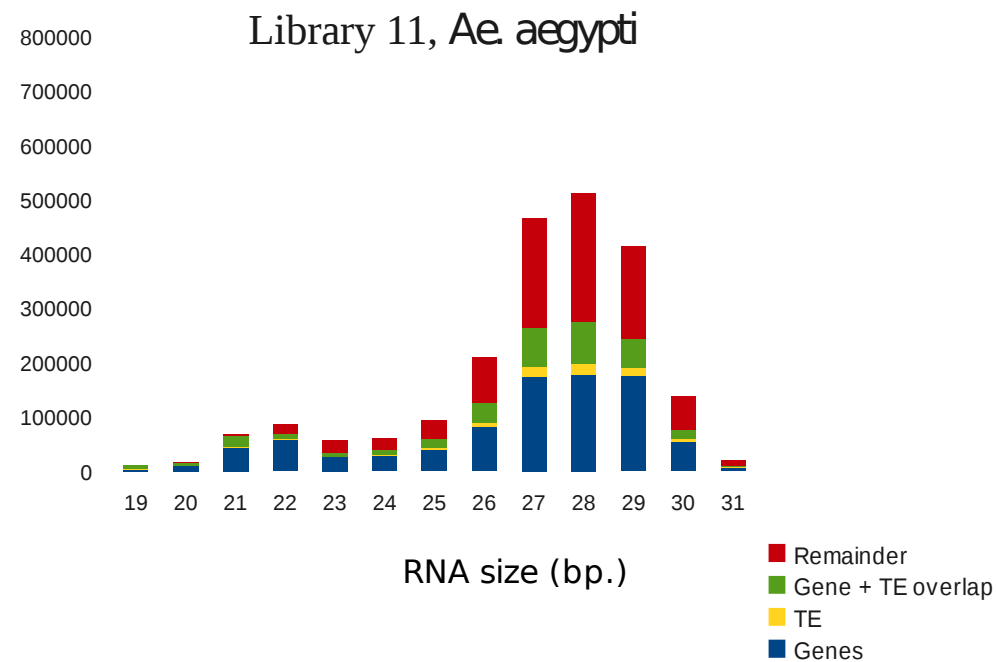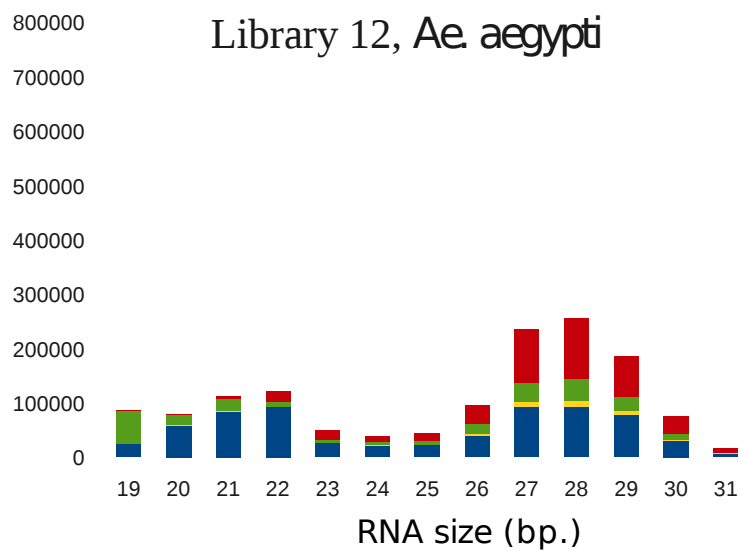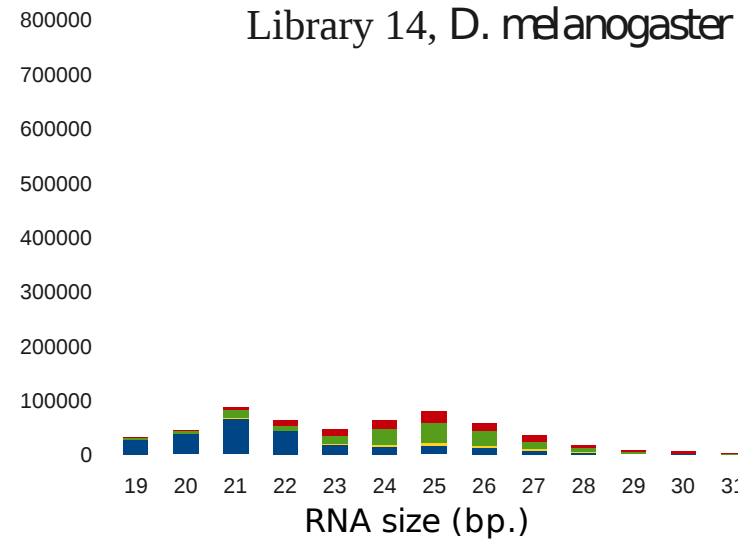

Supplement: Additional file 1 — Figure S1. Size distribution of Ae. aegypti small RNA abundance in each of the seven Ae. aegypti libraries and the single D. melanogaster library. The number of small RNAs mapping to Ae. aegypti genes, transposons, both, or neither, are shown as different colors for each size class (the legend is shown on the right). [file 1471-2164-12-606-S1.PDF]

# *AeBuster1*

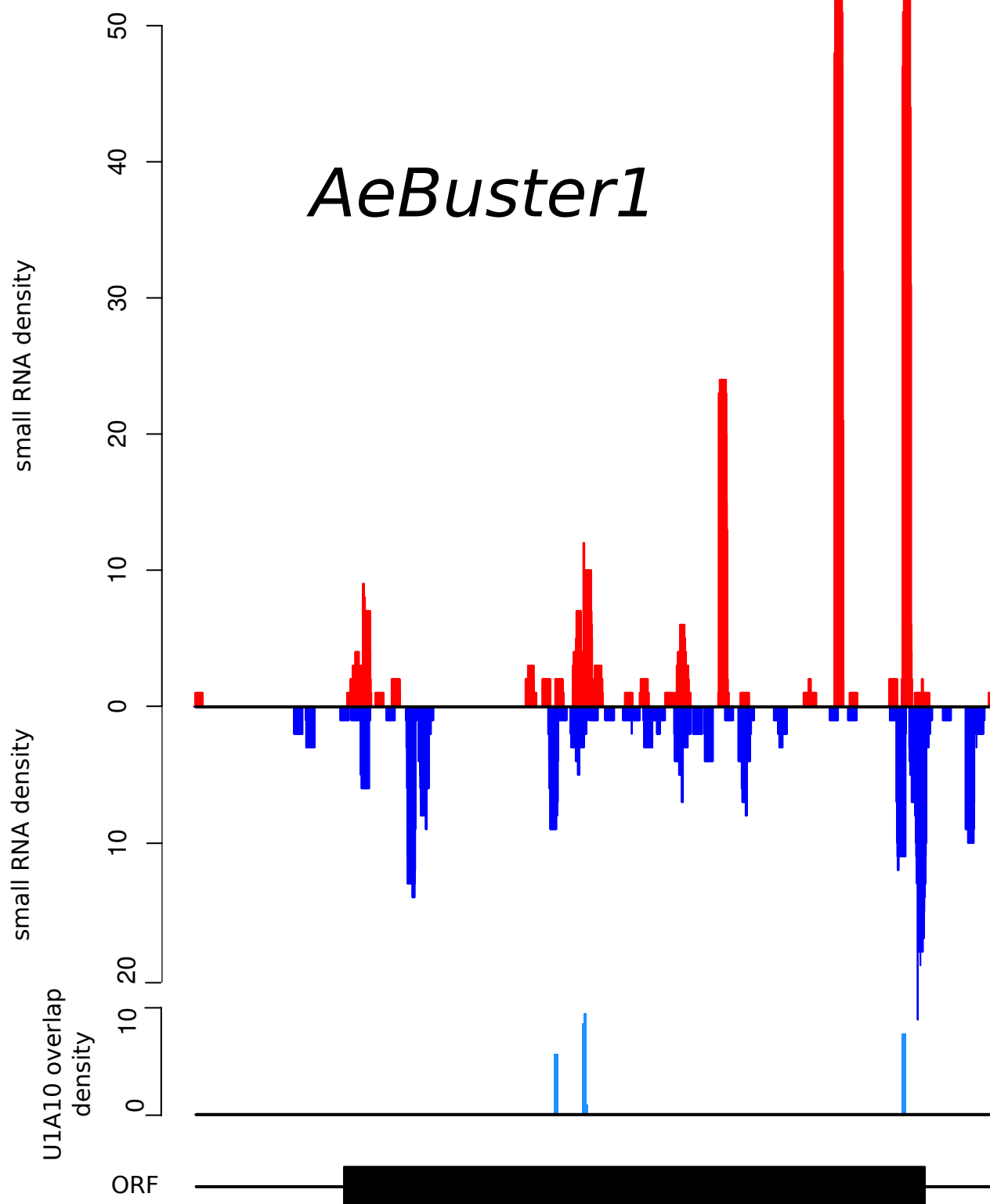

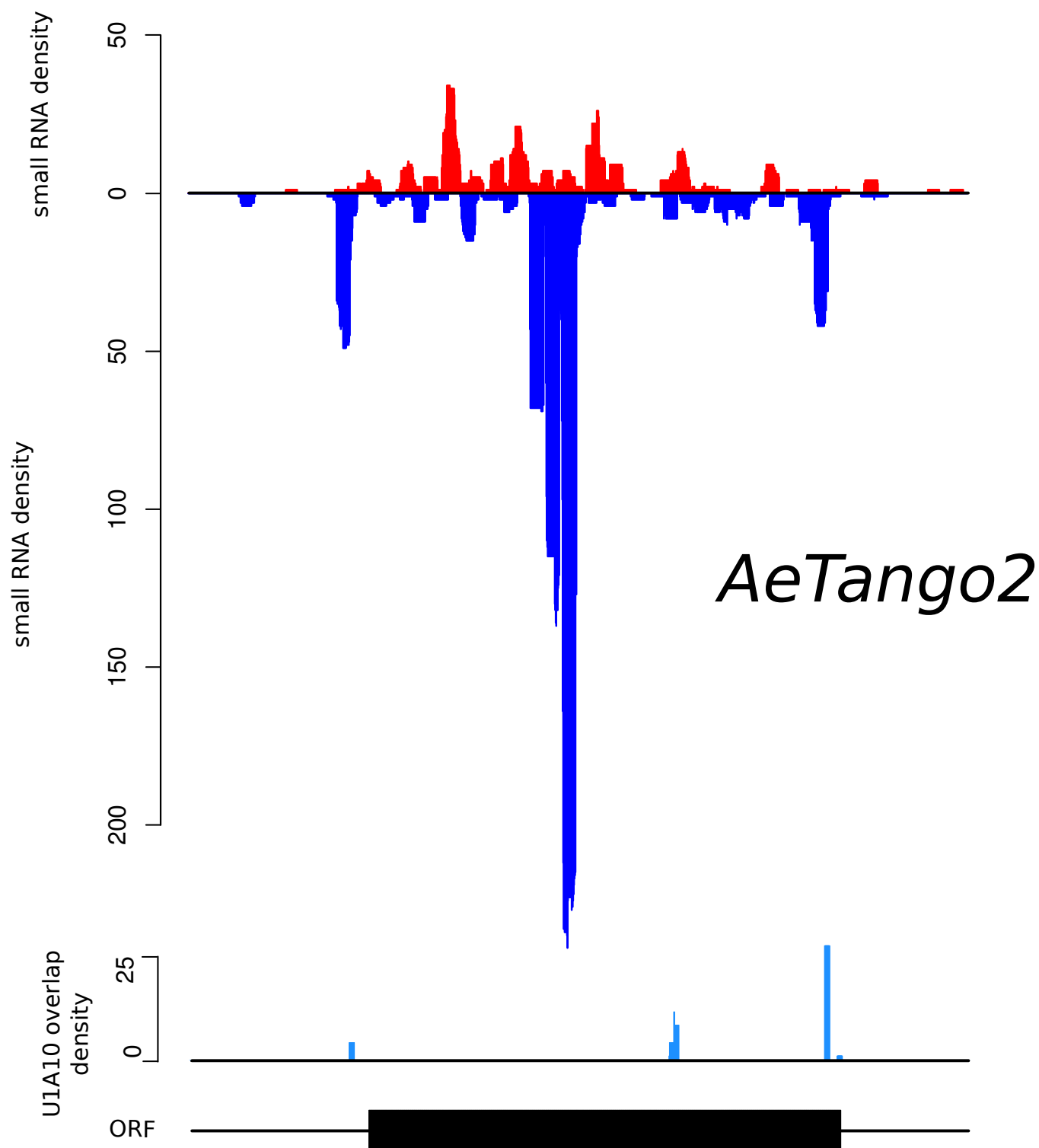

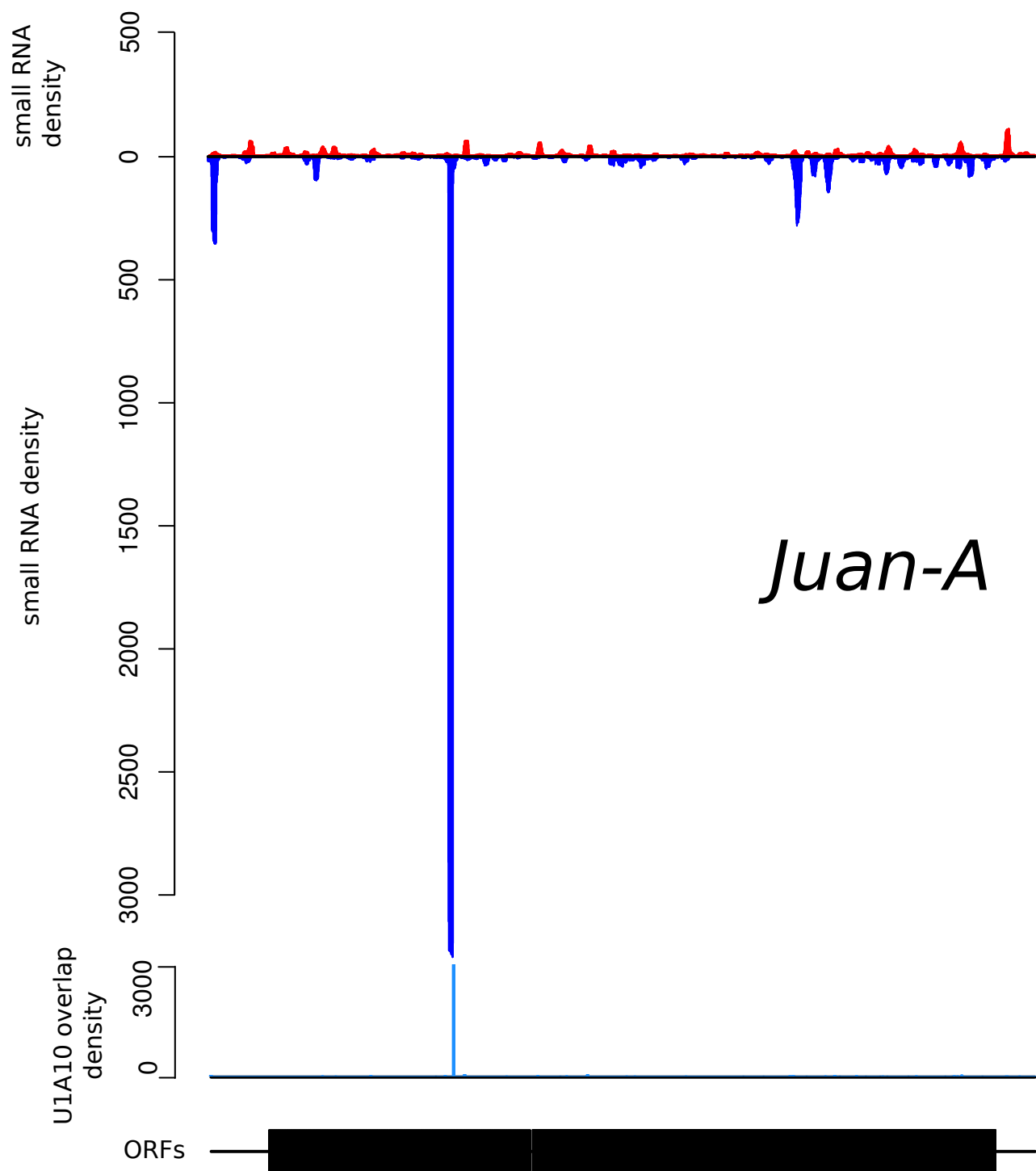

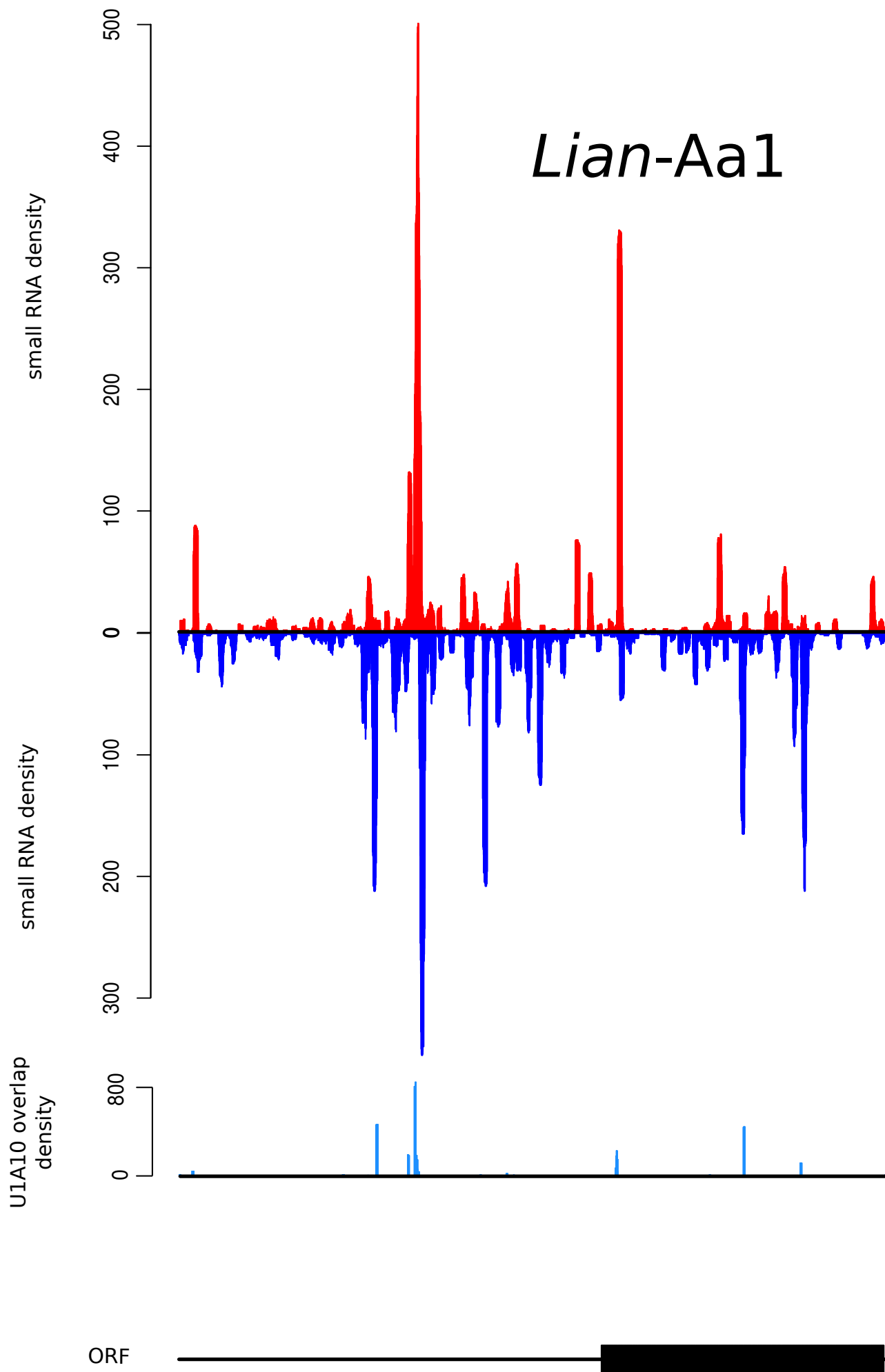

# *Mosqul-Aa2*

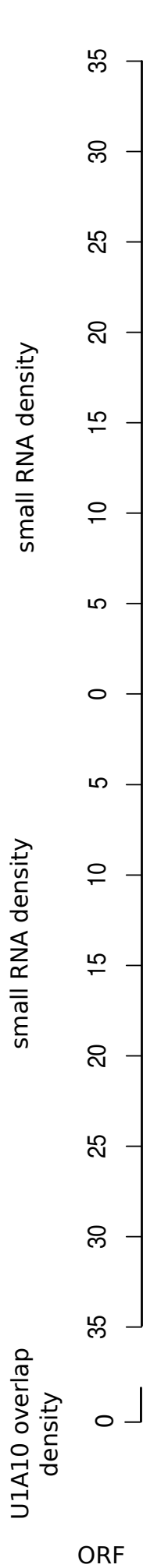

# *Pao\_Bel\_Ele1*

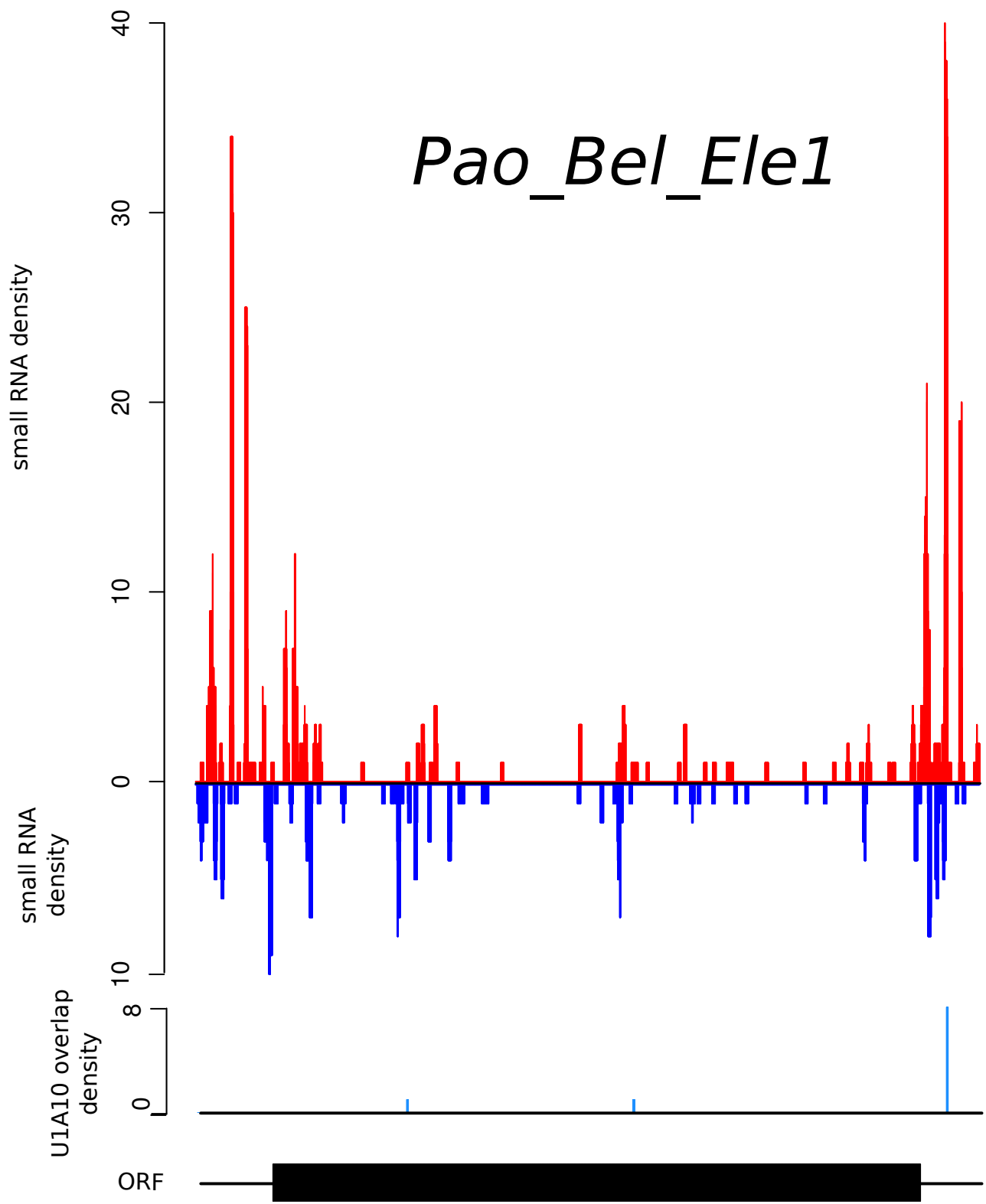

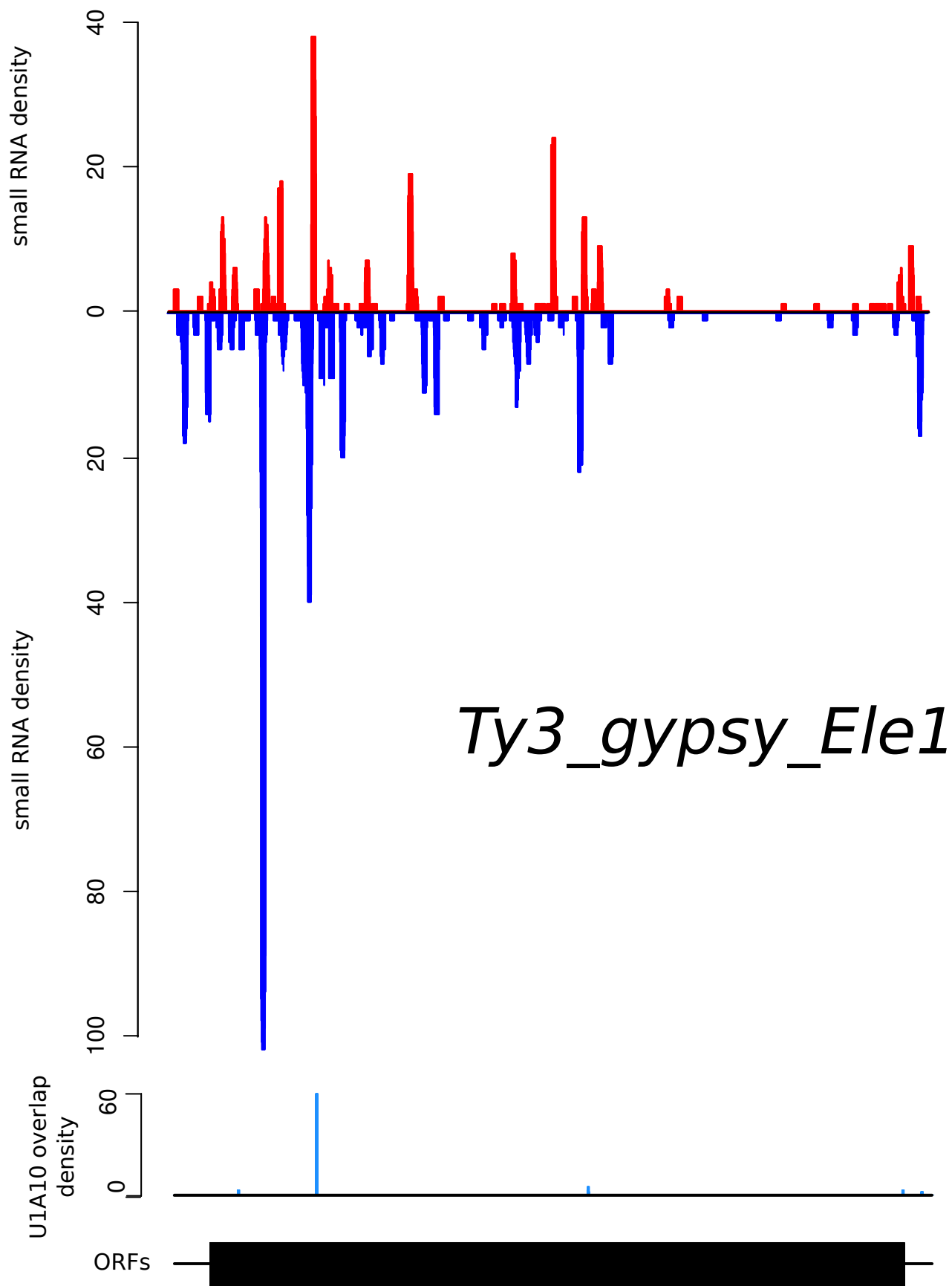

Supplement: Additional file 3 — Figure S1. Small RNA (>= 24 nt.) density plots for representative Ae. aegypti full length transposable elements. Small RNA density for all sequenced Ae. aegypti libraries mapping to the sense strand of the transposable element is show in red, mapping to the anti-sense strand shown in blue. Position and density of possible U1A10 overlap pairs is shown in light blue. Position of the ORF(s) is shown at the bottom of each figure. [file 1471-2164-12-606-S3.PDF]

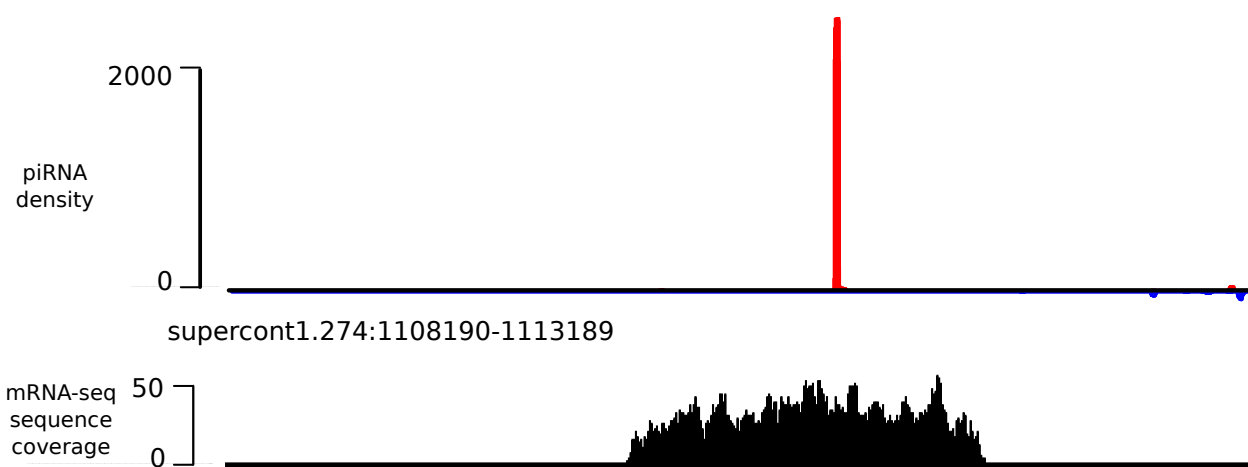

mRNA-seq transcript assembly

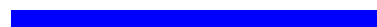

VectorBase gene

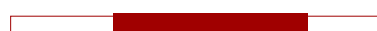

AAEL007686-RA

Supplement: Additional file 4 — Figure S1. piRNA density, mRNA-seq transcript coverage and assembly of the Ae. aegypti genomic region surrounding a putative Ae. aegypti homolog of the traffic jam gene. Genomic region supercontig identity and boundaries are shown below the piRNA density graph. mRNA-seq data were derived from an Ae. aegypti ovary tissue library. mRNA-seq transcript assembly, shown in blue, was performed using the CUFFLINKS program (Trapnell et al. 2010). Location on the genomic region of the mRNA transcript assembly and gene annotation, as reported in VectorBase, are show at the bottom. [file 1471-2164-12-606-S4.PDF]

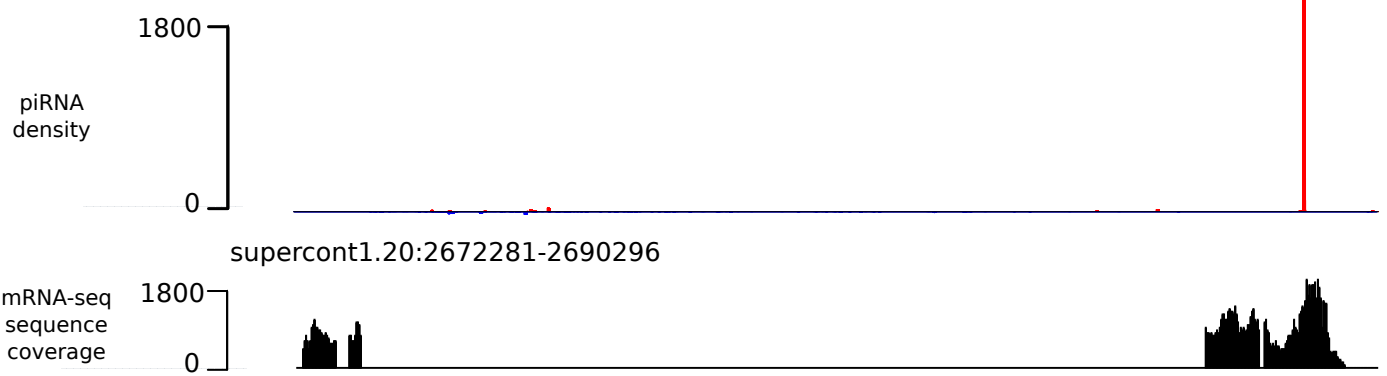

mRNA-seq transcript  
assembly

VectorBase genes

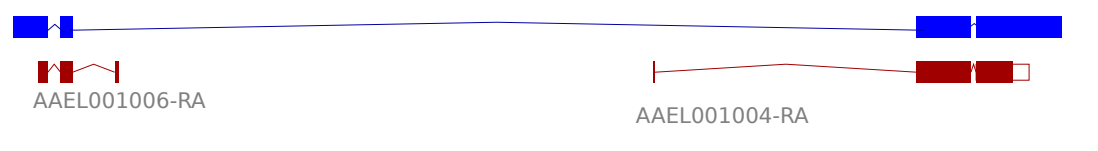

Supplement: Additional file 5 — Figure S1. piRNA density, mRNA-seq transcript coverage and assembly of the Ae. aegypti genomic region surrounding a putative Ae. aegypti homolog of the MAELSTROM gene. Genomic region supercontig identity and boundaries are shown below the piRNA density graph. mRNA-seq data were derived from an Ae. aegypti ovary tissue library. mRNA-seq transcript assembly, shown in blue, was performed using the CUFFLINKS program (Trapnell et al. 2010). Location on the genomic region of the mRNA transcript assembly and gene annotations, as reported in VectorBase, are show at the bottom. Reference cited in Additional file 5, Figure S1. 1. Trapnell et al., "Transcript assembly and quantification by RNA-Seq reveals unannotated transcripts and isoform switching during cell differentiation," Nature Biotechnology 28, no. 5 (2010): 511-515. [file 1471-2164-12-606-S5.PDF]
