# Supplementary material for: The mosquito Aedes aegypti has a large genome size and high transposable element load but contains a low proportion of transposon-specific piRNAs
Source: BMC Genomics. 2011 Dec 15;12:606. doi: 10.1186/1471-2164-12-606 (PMC3259105; doi:10.1186/1471-2164-12-606)
Supplement: Additional file 2 — Table S1. Number of piRNAs from Ae. aegypti libraries mapping to Transposable Element (TE) family consensus sequences and percentage occupancy of the genome by TE families. [file 1471-2164-12-606-S2.DOC]

**Additional File 1, Table S1.** Number of piRNAs from *Ae. aegypti* libraries mapping to Transposable Element (TE) family consensus sequences and percentage occupancy of the genome by TE families

(continued)

^1^ Data in this column was obtained from Arensburger et al. (2010)

^2^ Percentages were obtained by re-analysis of the Arensburger et al. (2010) data

Table S1. Continued.
